# Interspecies secreted surfactants induce emergent motility in *Pseudomonas aeruginosa*

**DOI:** 10.1101/2024.01.03.573969

**Published:** 2024-01-04

**Authors:** Delayna L. Warrell, Tiffany M. Zarrella, Christopher Machalek, Anupama Khare

**Affiliations:** a Laboratory of Molecular Biology, Center for Cancer Research, National Cancer Institute, National Institutes of Health, Bethesda, MD, USA; b Postdoctoral Research Associate Training Program, National Institute of General Medical Sciences, National Institutes of Health, Bethesda, MD, USA; c Current address: Department of Biology, Georgetown University, Washington, DC, USA

**Keywords:** *Pseudomonas aeruginosa*, *Staphylococcus aureus*, surfactants, motility, polymicrobial interactions, phenol-soluble modulins

## Abstract

In most natural environments, bacteria live in polymicrobial communities where secreted molecules from neighboring species alter bacterial behaviors including motility, but such interactions are understudied. *Pseudomonas aeruginosa* is a motile opportunistic pathogen that exists in diverse multispecies environments such as the soil and is frequently found in human wound and respiratory tract co-infections with other bacteria including *Staphylococcus aureus*. Here we show that *P. aeruginosa* can co-opt secreted molecules from other species for flagellar-based emergent motility. We found that in the presence of exoproducts from *S. aureus* and other bacteria, *P. aeruginosa* switched from swarming to an alternative form of motility where it slid and spread on semi-solid surfaces, and it showed similar behavior on hard agar where it was otherwise unable to move. Surfactants from these species were required for the *P. aeruginosa* emergent motility, and it was also induced by the addition of numerous exogenous biological and synthetic surfactants. Mutant analysis indicated that this motility was similar to a previously described mucin-based *P. aeruginosa* motility, ‘surfing’, albeit with divergent regulation. Thus, our study establishes a major *P. aeruginosa* surfing-like emergent motility where secreted surfactants from the host as well as neighboring bacterial and interkingdom species serve as public goods, allowing *P. aeruginosa* to access different environmental niches.

## INTRODUCTION

Bacteria are often found in dynamic, complex communities where interspecies secreted factors influence bacterial behaviors, such as antibiotic resistance [1–3], biofilm formation [4–7], and motility [8–10]. However, many of the polymicrobial interactions leading to the modification of these pathogenic traits have not been elucidated. The study of pairwise interactions has the potential to reveal behavioral changes that occur within communities as well as the underlying molecular mechanisms [11, 12].

Motility allows bacterial cells to disperse and migrate to new niches for nutrients, seek out prey, and adapt to new environments; thus, modulation of motility behaviors due to ecological interactions is likely to be advantageous for bacterial fitness. Several examples of changes in bacterial motility in mixed cultures compared to monocultures have been previously reported, including the inhibition of motility via secreted volatiles [9], and emergent motility in co-culture conditions where the monocultures did not move. These include social spreading between two non-motile species [13], exploration by non-motile bacteria upon sensing fungal volatiles [8], and non-motile bacteria inducing motility in a neighboring species, and then hitchhiking on the moving population [14].

*Pseudomonas aeruginosa* is a social, motile microbe that typically inhabits polymicrobial environments in soil, wounds, and persistent infections of the respiratory tracts of people with cystic fibrosis (CF) [15–17], in which the presence of *P. aeruginosa* is associated with worsened lung function and disease outcomes [18]. *P. aeruginosa* is commonly found in concert with *Staphylococcus aureus* in wound infections and nosocomial pneumonia, and these two species are the most frequently isolated bacterial pathogens from the airways of people with CF [19]. Chronic co-infections of *P. aeruginosa* and *S. aureus* are associated with increased disease severity [20–24]. Since these two organisms are often found together, we posited that interactions with *S. aureus* may alter *P. aeruginosa* pathogenic behaviors, including motility.

*P. aeruginosa* displays several well-established forms of motility: swimming, twitching, and swarming (Supp. Fig. 1A). Motility is intricately linked with virulence regulation, antibiotic resistance, and biofilm formation [25–27], and flagellar competency is associated with dissemination and pathogenesis [28–31]. During swimming motility *P. aeruginosa* uses a single polar flagellum to propel through liquid [32], while for twitching, type IV pili extension and retraction are required for movement on hard surfaces [33]. Swarming is a social behavior with a characteristic tendril formation on semi-solid surfaces that requires flagella and the secretion of rhamnolipids as a surfactant [34, 35]. Another type of motility that has been described is ‘surfing’, where *P. aeruginosa* spreads over semi-solid media containing the glycoprotein mucin, that is abundant in the mucus accumulated in the CF airways and gastrointestinal tract [36, 37]. This motility is thought to depend on the viscous nature and lubricant properties of mucin and requires *P. aeruginosa* flagellar function while pili and rhamnolipids are expendable [36, 38].

*P. aeruginosa* motility is known to be affected by interspecies interactions. Fungal volatiles farnesol and ethanol at high concentrations inhibit *P. aeruginosa* motility, while lower concentrations of ethanol induce swarming when carbon sources are limited, possibly signaling the availability of nutrients [39–41]. Further, it has been observed that *P. aeruginosa* engages in directional pili-based ‘exploratory motility’ in response to the *S. aureus* secreted cytotoxins phenol-soluble modulins (PSMs) [42]. *P. aeruginosa* explorers sense PSMs via the Pil-Chp chemosensory system to twitch on surfaces toward *S. aureus* [43]. However, how other *P. aeruginosa* motility behaviors are affected by *S. aureus* is not known.

In this study, we investigate whether additional *P. aeruginosa* motility behaviors are altered by *S. aureus* exoproducts. We observe that *P. aeruginosa* displays emergent motility in the presence of *S. aureus* secreted molecules, that enables it to slide and spread on semi-solid, and even hard agar surfaces where it is otherwise nonmotile. This motility depends on flagella, but does not require type IV pili, rhamnolipids, the three quorum sensing systems, or several other regulators that affect motility including the Gac/Rsm system. Further, transcriptomics of cells undergoing emergent motility did not reveal responses in motility genes, suggesting that the transition to emergent motility may not be due to transcriptional regulation. Multiple CF clinical isolates of *P. aeruginosa* and *S. aureus* also exhibit and induce emergent motility, respectively, indicating that these behaviors are widespread within these species. Further, secreted products from other bacterial species that produce biosurfactants, such as *Bacillus subtilis* and *P. aeruginosa* itself, but not from species that do not make this class of molecules, also promote emergent motility. Mutants that are deficient for the respective biosurfactants in these species are unable to induce emergent sliding and spreading, while diverse interkingdom biological and synthetic surfactants can, demonstrating that *P. aeruginosa* displays this motility in the presence of molecules with surfactant properties. Finally, a comparison with mucin-mediated surfing suggests that emergent sliding and spreading is a surfing-like motility, although movement on mucin requires the LasR quorum sensing regulator, while movement on surfactants is LasR-independent.

## RESULTS

### *P. aeruginosa* displays emergent motility in the presence of *S. aureus* secreted products.

To determine the effect of *S. aureus* secreted products on *P. aeruginosa* motility, we performed *P. aeruginosa* twitching, swimming, and swarming assays in the presence of 25% (v/v) *S. aureus* cell-free supernatant, or media salts as a control. As reported previously [42], we observed that *P. aeruginosa* twitched farther with the addition of *S. aureus* supernatant (Supp. Fig. 1B). In the swimming assay, while *P. aeruginosa* swam uniformly creating a circular swim area in the control agar, the swim patterns were irregular and oval shaped in the presence of *S. aureus* supernatant (Supp. Fig. 1C).

**Figure 1.**
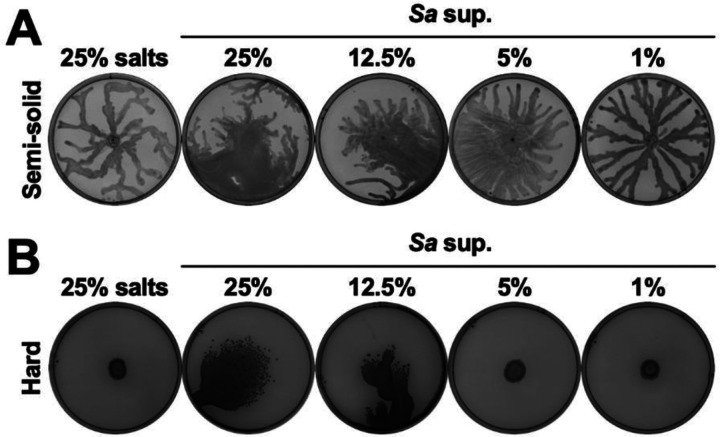
*S. aureus* secreted products induce *P. aeruginosa* emergent motility in a dose-dependent manner. *P. aeruginosa* was inoculated on **(A)** semi-solid or **(B)** hard agar plates containing the indicated percentages of media salts control or *S. aureus* supernatant, and plates were imaged after 24 hours incubation. Representative images of three independent replicates are shown. Additional replicates are shown in Extended Figure 1.

In a standard swarm assay on semi-solid agar plates (containing 0.5% agar) *P. aeruginosa* swarmed with characteristic tendril formation on the control plate (Fig. 1A). However, on plates with 25% *S. aureus* supernatant, *P. aeruginosa* exhibited a motility unlike swarming where the bacteria first slid on the agar and spread out across the plate, and then showed some tendril formation (Fig. 1A). *P. aeruginosa* exhibited similar motility on agar containing 12.5% *S. aureus* supernatant and an intermediate motility on 5% supernatant, while swarming resumed on plates with 1% supernatant (Fig. 1A). Next, we tested if this phenotype occurs on hard agar plates (containing 1.5% agar) in which *P. aeruginosa* is traditionally unable to move. While *P. aeruginosa* did not move on the hard agar control or on the plates containing 5% and 1% *S. aureus* supernatant (Fig. 1B), it exhibited emergent motility on the plates containing 25% and 12.5% *S. aureus* supernatant (Fig. 1B). Thus, *S. aureus* secreted products enable a distinct form of *P. aeruginosa* motility, even in conditions where *P. aeruginosa* is normally nonmotile.

### *P. aeruginosa* emergent motility requires flagella, but not pili or rhamnolipids.

The established forms of *P. aeruginosa* motility include swimming, which requires flagella [32], twitching, requiring pili [33], and swarming in which pili, flagella, and the surfactant rhamnolipids all contribute (Supp. Fig. 1A) [34, 35]. Additionally, motility in *P. aeruginosa* is also affected by quorum sensing systems and other regulatory pathways [34, 44, 45]. We therefore tested the requirement of flagella, pili, rhamnolipids, and known motility regulators for emergent sliding and spreading exhibited by *P. aeruginosa* in the presence of *S. aureus* secreted products.

**Figure 2.**
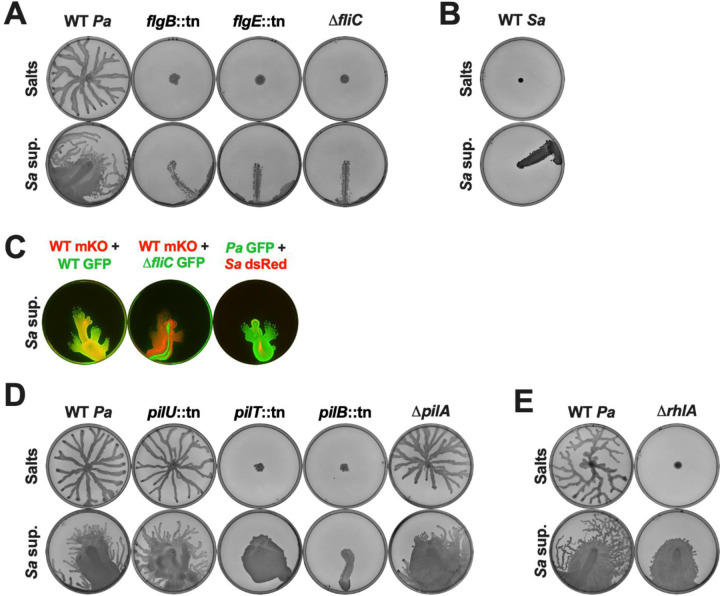
Emergent motility requires flagella, but not pili or rhamnolipids. The indicated strains of **(A,D,E)** *P. aeruginosa* or **(B)** *S. aureus* were inoculated on **(A,B,D,E)** semi-solid agar plates containing 25% media salts control or *S. aureus* supernatant as indicated. **(C)** From left to right, hard agar plates were inoculated with *P. aeruginosa* WT-mKO mixed with WT-GFP, *P. aeruginosa* WT-mKO mixed with Δ*fliC-*GFP, and *P. aeruginosa* WT-GFP mixed with *S. aureus* WT-dsRed. **(A–E)** Motility was imaged after 24 hours incubation. Representative images of three independent replicates are shown. Additional replicates are shown in Extended Figure 2.

First, we tested several mutants that lack the following structural components of the *P. aeruginosa* flagella: the proximal rod protein FlgB, the hook protein FlgE, or the flagellin subunit FliC [46], and are therefore defective in swimming (Supp. Fig. 2A). As expected, on the semi-solid control agar the WT was able to swarm while the flagellar mutants did not, and no motility was observed for all strains on the control hard agar (Fig. 2A and Supp. Fig. 2B). On the semi-solid and hard agar plates with *S. aureus* supernatant, the *flgB::*tn, *flgE::*tn, and Δ*fliC* mutants only slid on the plates without spreading out, unlike the motility exhibited by the WT strain (Fig. 2A and Supp. Fig. 2B). To examine the motility in a species that does not encode flagella, we tested *S. aureus* itself, and found that it was immobile on the control agar and slid on the plates with *S. aureus* supernatant like the *P. aeruginosa* flagellar mutants (Fig. 2B and Supp. Fig. 2C). To determine if these flagella-deficient strains could move in the presence of motile *P. aeruginosa*, these strains were fluorescently labeled, mixed, and added to hard agar with *S. aureus* supernatant. As expected, two distinctly labeled *P. aeruginosa* WT strains co-cultured on hard agar exhibited emergent motility equally (Fig. 2C). However, the Δ*fliC* mutant and *S. aureus* still only slid on the plate even in co-culture with *P. aeruginosa* WT cells that showed surface spreading (Fig. 2C), suggesting that this motility is an active process, and the presence of a motile population cannot compensate for loss of competent flagella. Hence, *P. aeruginosa* emergent motility in response to *S. aureus* secreted products requires active flagellar function.

We next determined if pili, which are required for twitching and contribute to swarming [34], are necessary for emergent sliding and spreading. We tested multiple twitching-deficient pili mutants (Supp. Fig. 3A) - two mutants deficient in pili retraction, *pilT*::tn and *pilU*::tn [47], two mutants lacking the extended pili fiber, *pilB*::tn, via deficiency in pili extension [48], and Δ*pilA* due to the lack of the pilin subunit [33], and two mutants deficient for pili-mediated chemotaxis, Δ*pilJ* and Δ*pilG* [49]. While these mutants showed differences in swarming on the control plates, all of these strains, except *pilB*::tn, displayed emergent motility on plates with *S. aureus* supernatant (Fig. 2D, Supp. Fig. 3B and C, Supp. Fig. 4A). The pili-extension mutant *pilB*::tn slid on the plate without spreading out, similar to the movement displayed by the flagellar mutants, (Fig. 2A), and there may be cross-regulation between pili and flagella [50, 51]. We therefore tested the pili mutants for swimming motility as a measure of their flagellar functionality. The *pilU*::tn and *pilB*::tn mutants had diminished swim rings, while the other mutants showed a normal swim phenotype (Supp. Fig. 3D). Since swimming requires flagella, it is likely that the flagellar dysfunction in the *pilB*::tn mutant also leads to a lack of emergent motility. In the *pilU*::tn strain, it is possible that the flagella is defective for swimming but still functional for emergent motility. Taken together, *P. aeruginosa* emergent motility in the presence of *S. aureus* secreted products requires flagella but not pili.

While swarming, *P. aeruginosa* secretes rhamnolipids, biosurfactants that lubricate the area and allow the cells to move over the surface [34, 35]. We found that while a mutant deficient in rhamnolipid production, Δ*rhlA*, was unable to swarm as previously reported [34, 35], it still slid and spread on both semi-solid and hard agar plates containing *S. aureus* cell-free supernatant (Fig. 2E and Supp. Fig. 4B), suggesting that rhamnolipids are not required for emergent motility.

Finally, to determine how this motility is regulated we analyzed the role of diverse *P. aeruginosa* signaling pathways in this motility. *P. aeruginosa* utilizes three quorum sensing systems: Las, Rhl, and PQS [52]. To examine the role of quorum sensing in this motility we tested mutant strains lacking each individually (Δ*rhlR*, Δ*lasR*, Δ*pqsA*), or in combination (Δ*rhlR* Δ*lasR* Δ*pqsA*). The Δ*rhlR*, Δ*lasR*, and Δ*rhlR* Δ*lasR* Δ*pqsA* mutants did not swarm on the control plates, while the Δ*pqsA* mutant did (Supp. Fig. 5A). However, all mutants displayed emergent motility on the plates with *S. aureus* supernatant to varying degrees, with the Δ*pqsA* strain being hyper-motile compared to the other strains and covering the entire semi-solid agar plate (Supp. Fig. 5A and B). We also tested mutants from additional regulatory pathways that control motility: Δ*gacA*, response-regulator of the Gac/Rsm pathway involved in virulence and motility; Δ*rsmA*, a posttranscriptional regulator of the Gac/Rsm pathway; Δ*retS*, a response regulator which can block GacA function [53]; Δ*wspR*, the response regulator of the Wsp chemosensory pathway [54, 55]; and Δ*toxR*, a c-di-GMP binding protein involved in motility and biofilm regulation [56]. The Δ*retS* and Δ*rsmA* mutants did not swarm, while the Δ*gacA*, Δ*wspR*, and Δ*toxR* mutants exhibited swarming similar to WT *P. aeruginosa* (Supp. Fig. 6). Despite these differences in swarming, all these regulatory mutants exhibited emergent sliding and spreading (Supp. Fig. 6). Therefore, these regulatory pathways are not required for *P. aeruginosa* emergent motility, although quorum sensing may affect motility patterns.

### Cells undergoing emergent motility do not transcriptionally regulate motility pathways.

**Figure 3.**
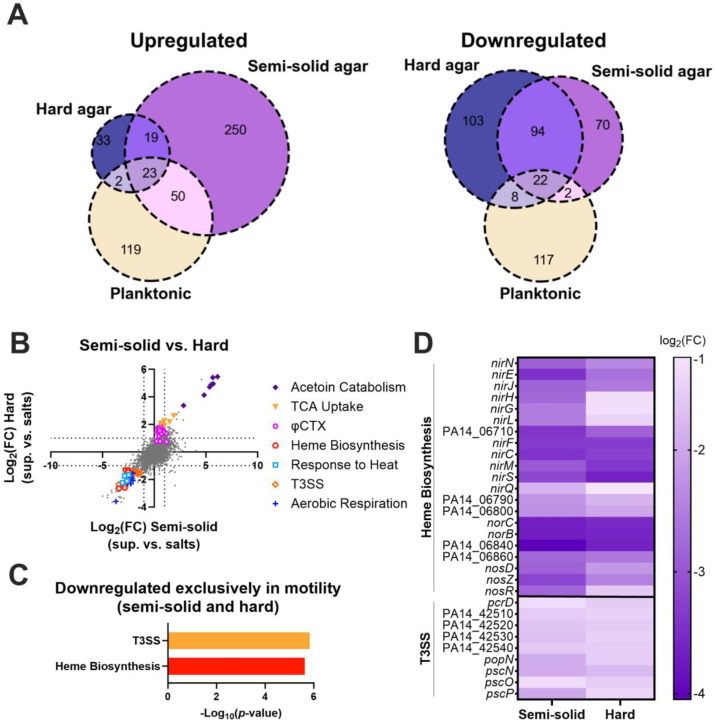
*P. aeruginosa* differentially regulates genes involved in T3SS, and heme biosynthesis, but not motility, during emergent motility on *S. aureus* secreted products. Transcript levels were compared between *P. aeruginosa* cells exposed to *S. aureus* supernatant and those exposed to media salts control on semi-solid and hard agar after 17 hours. **(A)** Venn diagrams of upregulated and downregulated genes on hard and semi-solid agar, as well as in planktonic *P. aeruginosa* cells after *S. aureus* supernatant exposure compared to media salts control [57]. **(B)** Scatter plot of log_2_(fold-change) transcript levels on hard agar compared to the log_2_(fold-change) of transcript levels on semi-solid agar. Differentially regulated genes in pathways enriched in the common downregulated genes, as well as in TCA uptake, acetoin catabolism, and φCTX are shown. **(C)** GO enrichment of *P. aeruginosa* genes differentially expressed on both semi-solid and hard agar plates, but not in planktonic phase [58–60]. Nonredundant categories are shown. **(D)** Heatmap showing the log_2_(fold-change) of genes associated with enriched pathways for heme biosynthesis and T3SS on semi-solid and hard agar.

To identify which pathways are differentially regulated during emergent sliding and spreading, we performed RNA sequencing (RNA-seq) on *P. aeruginosa* cells scraped from the leading edge on semi-solid or hard agar plates containing the media salts control or *S. aureus* supernatant at 17 hours (Supp. Fig. 7A). We found approximately 100 to 350 genes that were upregulated and downregulated on the supernatant compared to the media salts control in both cases (Supp. Tables 1 and 2). We reasoned that the differentially regulated genes would include genes specific to this motility as well as genes that were regulated upon exposure to unrelated *S. aureus* secreted molecules. We therefore compared the genes to those we previously identified as being differentially regulated in early log-phase *P. aeruginosa* planktonic cultures 2 hours after the addition of *S. aureus* supernatant compared to the addition of media salts as a control [57], and found significant overlaps (Fig. 3A). Some of the highest upregulated genes in both motility conditions were genes involved in tricarboxylic acids (TCA) uptake and acetoin catabolism, which are upregulated in planktonic conditions as well, likely due to *S. aureus* secreted citrate and acetoin, respectively [57] (Fig. 3B, Supp. Fig. 7B and C, Supp. Tables 2, 3, and 4). Next, we performed Gene Ontology (GO) enrichment analysis of the *P. aeruginosa* genes differentially regulated in the individual motility conditions, both motility conditions (semi-solid and hard agar plates), as well as those regulated exclusively in both motility conditions, but not in the planktonic phase [58–60] (Fig. 3C, Supp. Fig. 7D and E, and Supp. Tables 3, 4, and 5). Genes involved in aerobic respiration and response to heat were enriched in the genes downregulated in both motility conditions (Supp. Fig. 7E and Supp. Table 5), but these genes were also downregulated in the planktonic phase and are likely unrelated to the motility (Supp. Fig. 7E and Supp. Tables 4 and 5).

The *P. aeruginosa* genes differentially regulated exclusively in both motility conditions, but not in the planktonic phase, were not enriched for any motility pathways (Fig. 3C and Supp. Table 5), suggesting that transcriptional regulation may not play an important role during emergent motility, consistent with our results that major motility regulators are not required for this motility (Supp. Fig. 5 and 6). Instead, genes downregulated exclusively in the motility conditions showed enrichment of the Type 3 Secretion System (T3SS) and heme biosynthesis pathways, while the upregulated genes showed no statistically enriched pathways (Fig. 3C and D, and Supp. Table 5). Additionally, on the hard agar there was an upregulation in transcripts from the phage φCTX (Fig. 3B, Supp. Fig. 7B, and Supp. Table 2), indicating prophage induction in this condition. This likely explains the presence of plaque-like clearings on the hard agar (e.g. in Fig. 1B), which was especially apparent for the LasR mutant (Supp. Fig. 5). Multiple cellular stresses can lead to both induction of prophage and downregulation of T3SS [61–64], indicating that *P. aeruginosa* senses physiological stress while undergoing emergent motility. Further, heme biosynthesis and associated denitrification genes (e.g. *nirS*, *nirQ*, *norC, norB)* are induced in anaerobic conditions [65]. The relative downregulation of these genes during emergent motility may be indicative that the control cells (swarming cells in the semi-solid agar and non-motile cells in the hard agar) are switching to anaerobic metabolism, while cells undergoing emergent motility continue with aerobic respiration. Thus, *P. aeruginosa* may experience physiological stress and metabolic changes while undergoing emergent motility in the presence of *S. aureus* exoproducts.

### Interspecies surfactants facilitate emergent motility in *P. aeruginosa*.

To determine the generality of this emergent motility, we performed the motility assay with clinical isolates of both species. We tested four clinical isolates of *P. aeruginosa* collected from different people with CF (CF017, CF033, CF057, and CF095) (Supp. Table 6) on both semi-solid and hard agar and observed that the isolates CF017 and CF057 swarmed and exhibited emergent motility, while isolates CF033 and CF095 did not (Supp. Fig. 8A and B). To determine if the motility deficiencies were due to the dysfunction of motility appendages, we tested these isolates for swimming and twitching. Isolate CF017 swam similar to *P. aeruginosa* PA14, while isolates CF033, CF057, CF095 all had swimming deficiencies (Supp. Fig. 9A). Additionally, the twitch zone of CF033 was similar to PA14, but CF017 and CF057 had smaller twitch zones and CF095 did not twitch (Supp. Fig. 9B). Thus, the isolates that do not exhibit the emergent motility may have flagellar and/or pili deficiencies.

These *P. aeruginosa* strains were co-isolated with four *S. aureus* strains, CF019, CF032, CF058, and CF100, respectively (Supp. Table 6). Cell-free supernatants from each of these *S. aureus* clinical isolates could induce emergent motility in *P. aeruginosa* PA14 (Supp. Fig. 10A and B). These results demonstrate that while there is some variation, clinical strains of *P. aeruginosa* and *S. aureus* can exhibit and induce emergent surface spreading, respectively.

**Figure 4.**
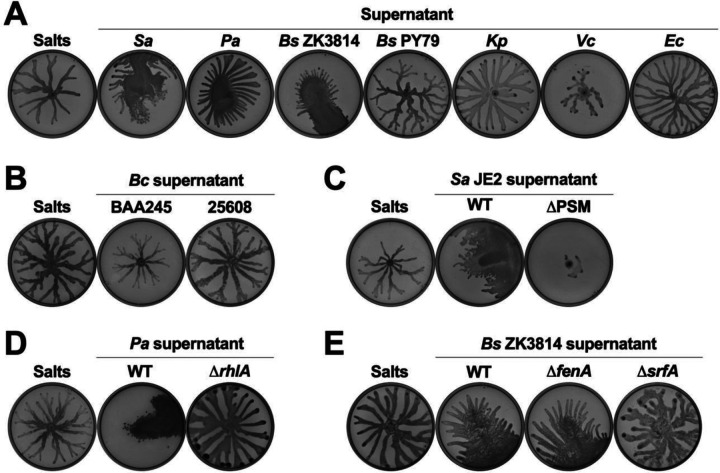
Interspecies secreted surfactants induce emergent motility in *P. aeruginosa*. **(A–E)** *P. aeruginosa* was inoculated on semi-solid agar plates containing 25% media salts control or supernatant from the indicated species: *S. aureus* (JE2), *P. aeruginosa* (PA14), *B. subtilis, K. pneumoniae, V. cholerae, E. coli* (MG1655), and *B. cenocepacia*. Supernatant was from **(A, B)** WT strains and **(C–E)** mutants for the biosynthesis of surfactants. Images were taken after 24 hours incubation. Representative images of three independent replicates are shown. Additional replicates are shown in Extended Figure 4.

Next, we investigated whether other species induce this emergent motility, and found that *P. aeruginosa* exhibited this motility on plates with supernatant from *S. aureus, P. aeruginosa*, and *Bacillus subtilis* ZK3814 (Fig. 4A), but swarmed on plates with supernatant from *B. subtilis* PY79, *Klebsiella pneumoniae, Vibrio cholerae, Escherichia coli, Burkholderia cenocepacia* BAA245, and *B. cenocepacia* 25608 (Fig. 4A and B).

A common class of molecules known to be secreted by each of the inducing strains, but none of the non-inducing strains, and previously implicated in motility, is biosurfactants [66]. *S. aureus* produces phenol soluble modulins (PSMs) [67–69], *P. aeruginosa* produces rhamnolipids [70], and *B. subtilis* ZK3814 produces fengycin and surfactin [71]. We therefore tested the supernatant of mutants deficient in each of these biosurfactants for their ability to induce motility in *P. aeruginosa*. The supernatants of the *S. aureus* JE2 Δ*psmɑδ*, which is deficient in the production of PSMs ɑ1–4 and delta toxin, as well as a Δ*psmɑβδ* mutant (that lacks all PSMs) in the *S. aureus* LAC strain, did not induce emergent motility in *P. aeruginosa* (Fig. 4C and Supp. Fig. 11A, B, and C). Further, supernatant from *agrA*::tn, *agrB*::tn, or *agrC*::tn, mutants of the *S. aureus agr* quorum sensing system which is required for the production of PSMs, also did not lead to *P. aeruginosa* emergent motility (Supp. Fig. 11A and D). Similarly, we found that *P. aeruginosa* did not exhibit this motility on plates containing supernatant from the *P. aeruginosa* Δ*rhlA* mutant lacking the biosurfactant rhamnolipids (Fig. 4D and Supp. Fig. 12A). Finally, for *B. subtilis*, supernatant from Δ*srfA*, a mutant deficient for surfactin production, was unable to induce *P. aeruginosa* emergent motility, unlike the parental WT *B. subtilis* ZK3814 as well as Δ*fenA*, a mutant deficient for fengycin production (Fig. 4E and Supp. Fig. 12B). Collectively, these data show that the production of biosurfactants such as *S. aureus* PSMs, *P. aeruginosa* rhamnolipids, and *B. subtilis* surfactin, is required for the interspecies induction of sliding and spreading motility in *P. aeruginosa*.

### Detergents and surfactants are sufficient to induce emergent motility in *P. aeruginosa*.

**Figure 5.**
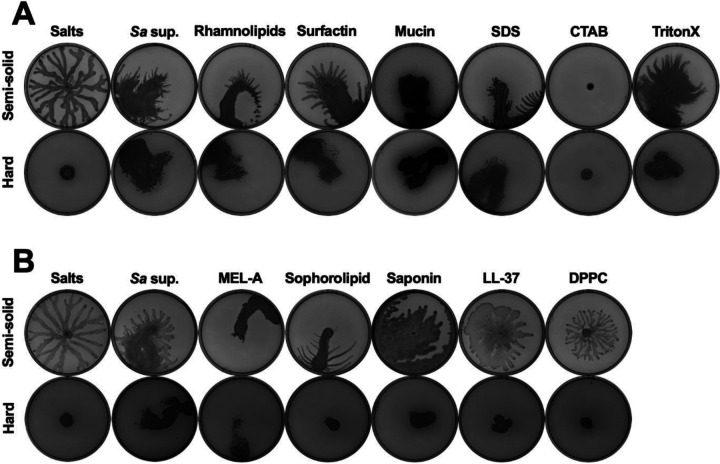
The addition of diverse biotic and synthetic surfactants is sufficient to induce emergent motility in *P. aeruginosa*. **(A,B)** *P. aeruginosa* was inoculated on **(top)** semi-solid or **(bottom)** hard agar plates containing **(A)** (from left to right) 25% media salts control or *S. aureus* supernatant, or 25% media salts with the addition of 50 μg·mL^−1^ rhamnolipids, 5 μg·mL^−1^ surfactin, 0.4% mucin, 0.1% SDS, 0.1% CTAB, or 0.1% Triton X-100; or **(B)** 25% media salts control or *S. aureus* supernatant; or 25% media salts with the addition of 25 μg·mL^−1^ MEL-A, 25 μg·mL^−1^ sophorolipid, 25 μg·mL^−1^ saponin, 100 μg·mL^−1^ LL-37, or 100 μg·mL^−1^ DPPC. **(A,B)** Images were taken after 24 hours incubation. Representative images of three independent replicates are shown. Additional replicates are shown in Extended Figure 5.

Given that bacterial-secreted surfactants were necessary to induce emergent motility in *P. aeruginosa*, we investigated if the addition of biological and synthetic surfactants was sufficient to induce this motility. We tested commercially obtained biosurfactants from the motility-inducing species, including rhamnolipids from *P. aeruginosa* and surfactin from *B. subtilis* ZK3814; mucin, which has been previously implicated in *P. aeruginosa* surfing motility [38]; and the synthetic surfactants sodium dodecyl sulfate (SDS), cetrimonium bromide (CTAB), and Triton X-100. *P. aeruginosa* exhibited emergent motility, to varying degrees, on plates with *S. aureus* supernatant, the biosurfactants surfactin, rhamnolipids, and mucin, and the synthetic surfactants SDS and Triton X-100, but motility was inhibited on both semi-solid and hard agar plates with the addition of CTAB (Fig. 5A). Further, we tested biosurfactants from interkingdom species, such as mannosylerythritol lipid A (MEL-A) and sophorolipid from yeasts, plant-produced saponin, and human cathelicidin LL-37 and pulmonary surfactant dipalmitoylphosphatidylcholine (DPPC). All these interkingdom surfactants, except DPPC, induced the sliding and spreading motility, especially on semi-solid agar (Fig. 5B). Thus, diverse biological and synthetic surfactants are sufficient to induce *P. aeruginosa* emergent motility.

### *P. aeruginosa* surfactant-induced surface spreading is a surfing-like motility.

Previously, it was reported that *P. aeruginosa* exhibits ‘surfing’, a surface-associated, flagellar-based motility which occurs on semi-solid agar containing mucin [36, 38, 72, 73]. To compare the genetic requirements in *P. aeruginosa* of mucin-based surfing behavior to microbial surfactant-induced emergent motility, we tested motility appendage, quorum sensing, and other regulatory mutants on semi-solid plates containing *S. aureus* supernatant or mucin. The appendage mutants showed similar phenotypes for motility on *S. aureus* supernatant and mucin, where *flgB*::tn, Δ*fliC*, and *pilB*::tn showed no motility on either, while *pilT*::tn and Δ*pilA* moved on both, though *pilT*::tn moved only moderately on mucin (Supp. Fig. 13A).

Previously, Rhl and Las as well as components of Gac/Rsm were reported to contribute to mucin-based surfing [36, 72, 73]. We observed that while the Δ*pqsA* mutant moved on both the *S. aureus* and mucin plates, the Δ*rhlR* and Δ*rhlR* Δ*lasR* Δ*pqsA* mutants moved moderately on *S. aureus* supernatant and had no movement on the plates with mucin (Supp. Fig. 13B). Interestingly, the Δ*lasR* mutant moved on the *S. aureus* supernatant but not on mucin, demonstrating a divergence of quorum sensing requirements for mucin-based surfing [36, 38] and surfactant-dependent motility. The Δ*retS*, Δ*gacA*, Δ*rsmA*, Δ*wspR*, and Δ*toxR* regulatory mutants all moved on both *S. aureus* supernatant and mucin, but Δ*retS* and Δ*rsmA* had less movement on both plate types (Supp. Fig. 14).

**Figure 6.**
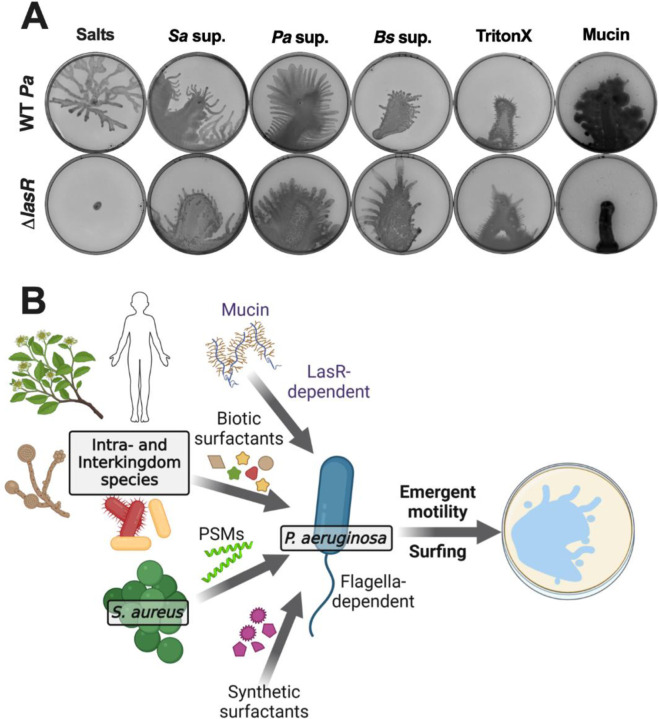
The Las system is required for surfing motility on mucin, but not on surfactants. **(A)** *P. aeruginosa* WT and Δ*lasR* were inoculated on semi-solid agar plates containing (from left to right) 25% media salts control or supernatant from the indicated species: *S. aureus* (JE2), *P. aeruginosa* (PA14), or *B. subtilis* (ZK3814); or 25% media salts with the addition of 0.1% Triton X-100 or 0.4% mucin. Images were taken after 24 hours incubation. Representative images of three independent replicates are shown. Additional replicates are in Extended Figure 6. **(B)** Working model of *P. aeruginosa* motility in response to exogenous surfactants. The glycopeptide mucin, PSMs secreted from *S. aureus*, and other biotic surfactants secreted by human cells, plants, yeast, and bacteria, as well as synthetic surfactants, are sensed by *P. aeruginosa*. In response, *P. aeruginosa* exhibits flagella-dependent emergent motility on surfaces. In contrast to the motility in the presence of surfactants, mucin-mediated surfing motility requires the LasR quorum sensing system in *P. aeruginosa*.

To further delineate the role of LasR in motility on a variety of surfactants, we tested the Δ*lasR* mutant on semi-solid plates with supernatants from *S. aureus*, *P. aeruginosa*, and *B. subtilis* ZK3814, as well as with the addition of mucin and the surfactant Triton X-100. While the Δ*lasR* mutant did not move on mucin, it exhibited the motility on all of the other supernatant or surfactant-containing plates (Fig. 6A). Overall, the surfactant-dependent emergent motility we observed and mucin-dependent surfing motility have similar appendage and regulatory requirements, indicating that surfactant-based emergent sliding and spreading is a surfing-like motility where the exact quorum-sensing regulation may differ between movement on mucin compared to other surfactants.

## DISCUSSION

Bacterial locomotion is an advantageous trait that enables migration to more favorable environments and access to fresh nutrients. Within microbial communities, interspecies secreted factors can affect bacterial motility, by activation [8], inhibition [9], or endowing nonmotile strains the capability to move [13, 14]. Many of these observations were reported by examining pairwise interactions, which can reveal novel behavioral phenomena as well as the underlying mechanisms [11]. Here, we interrogated how *P. aeruginosa* motility is affected by *S. aureus* secreted molecules and uncovered a surfing-like motility that depends on the presence of exogenous interspecies or synthetic surfactants (Fig. 6B). The surfactant-based motility we observed requires flagellar function, but not type IV pili, rhamnolipids, quorum sensing, or other known motility regulatory systems like Gac/Rsm. It permits emergent motility when *P. aeruginosa* is typically nonmotile and is induced by surfactants from a variety of species, including co-infecting pathogens, putative environmental neighbors, as well as infection hosts, demonstrating that *P. aeruginosa* can take advantage of specific cues from nearby species to explore new niches.

We observed that in the presence of interspecies surfactants, *P. aeruginosa* cells first slid on the agar surface similar to nonmotile bacteria, like *S. aureus* or non-flagellated *P. aeruginosa* strains (Fig. 2A and B). The cells then spread over the surface, an active process that required flagellar function. This was followed by tendril formation on semi-solid agar, or likely dispersal of individual cells on hard agar that resulted in colonies at the mobile leading edge. The motility was induced by the presence of diverse types of biological and synthetic molecules of a variety of chemical compositions, suggesting that their shared properties of reducing surface tension and acting as wetting agents led to the motility. The reduced surface tension likely allows for increased movement, resulting in motility even on hard agar, where *P. aeruginosa* is otherwise nonmotile, and enabling surface motility without rhamnolipid production, unlike what is seen for swarming. Intrinsic rhamnolipid production occurs and is required during swarming, while the presence of exogenous rhamnolipids leads to emergent motility (Fig. 5A). Given that *P. aeruginosa* rhamnolipid production is quorum-sensing dependent and requires high cell density, it is likely that in the presence of foreign surfactants, *P. aeruginosa* cells immediately sense the exogenous surfactants resulting in emergent motility before they produce rhamnolipids.

Motility with similar characteristics has been previously reported in *P. aeruginosa*. When other gelling agents, the polysaccharides gellan gum and carrageenan, are used in place of agar in semi-solid media, *P. aeruginosa* exhibits flagellar-dependent, rhamnolipid-independent surface motility due to the reduction in surface tension [74], like the surfactant-mediated motility we observed. Further, surfing motility in *P. aeruginosa* has previously been described to occur on semi-solid agar plates (0.5 – 1.0% agar) containing the glycopeptide mucin and require the use of flagella [36, 38]. While these motility types were thought to be distinct [36, 38, 74], our data suggests that these are likely variations of the same *P. aeruginosa* surface motility that occurs upon reduction of surface tension and requires active flagella, but not pili or rhamnolipids, where the main distinction is that only mucin-mediated surfing requires LasR quorum sensing (Fig. 6A) [36, 38, 74]. We propose that the term ‘surfing’ encompasses all these variations, and our study thus establishes surfing as a major form of *P. aeruginosa* motility that occurs on both semi-solid and hard surfaces in the presence of a variety of biotic and abiotic surfactants but is distinct from other established motility types like swimming, twitching, or swarming.

The signal transduction pathway from sensing environmental changes brought on by surfactants leading to flagellar motility remains elusive. We examined the requirement of several regulators that control motility [75], such as components of Pil-Chp, quorum sensing, Gac/Rsm, and Wsp surface sensing, but none were needed for surfactant-based surfing. Nonetheless, there were noticeable differences in the appearance of quorum sensing mutant surfers. On semi-solid agar, the final step of tendril formation was absent in Rhl mutants that are expected to lack rhamnolipid production, indicating that the tendrils here may be similar to those seen in swarming, and require intrinsic production of rhamnolipids. In addition, mutants devoid of the PQS pathway, which plays a role in swarming tendril repulsion [76], are hyper-motile on semi-solid agar, suggesting that the PQS pathway may play a role in limiting surfactant-based surfing. Given the non-essentiality of these pathways for surfing, it is possible that the *P. aeruginosa* polar flagellum directly senses the surfactant by the alleviation of impediments on flagellar rotation [77]. Moreover, many of the signaling cascades that lead to changes in flagellar motion involve the motile-to-sessile second messenger cyclic di-GMP [54], which is a putative target for future exploration of the mechanisms underlying this interspecies-induced motility.

*P. aeruginosa* and *S. aureus* often co-infect wounds and the airways of people with CF where they are associated with faster lung function decline, an increase in intravenous antibiotic treatments, and an increase in lower airway inflammation [20, 21]. We observed that the presence of *S. aureus* PSMs, which are amphipathic alpha-helical peptides with surfactant properties, induces surfing motility in *P. aeruginosa*. Previously, these *S. aureus* secreted toxins have been implicated in enabling motility in both species. In *S. aureus*, PSMs allow nonmotile *S. aureus* to slide on surfaces [68]. Additionally, *P. aeruginosa* senses PSMs via the Pil-Chp chemosensory system to induce pili-dependent directional ‘exploratory motility’ [42, 43], which is a motility distinct from the one we describe. *P. aeruginosa* explorers require pili and components of the Pil-Chp system, such as the chemoreceptor PilJ [43], which are not necessary for flagellar-dependent surfactant-based surfing. Sensing of surface hardness and viscosity plays a role in stimulating motility behaviors [78, 79], with twitching and swarming occurring on hard and semi-solid surfaces, respectively. However, we observed that even on hard agar surfactant-based surfing still solely required flagella and not pili, in contrast to the directionality of exploratory motility detected on hard agar [42, 43]. PSMs produced by neighboring *S. aureus* cells are also thought to alter the flow of *P. aeruginosa* rhamnolipids, leading to repulsion of *P. aeruginosa* swarm tendrils [80]. Thus, there is an overarching role for PSMs in inducing and altering motility in *P. aeruginosa*, yet the mode of locomotive response likely differs based on additional environmental stimuli and/or how these molecules are sensed by *P. aeruginosa*.

In addition to *S. aureus*-produced PSMs [68], many microbes secrete diverse classes of biosurfactants that enable locomotion [81], such as the lipopeptide surfactin by *Bacillus subtilis* [82], the glycolipids rhamnolipids by *Pseudomonas aeruginosa* and *Burkholderia cenocepacia* [83, 84], and the aminolipid serrawettin by *Serratia* spp. [85]. As secreted factors, interspecies surfactants can serve as public goods and enable other species to co-opt these wetting agents for motility, and we observed that *P. aeruginosa* can utilize diverse intra- and interkingdom surfactants for surfing. In phyllosphere-dwelling bacterial species, secretion of the surfactant viscosin B by *Pseudomonas* sp. FF1 leads to co-swarming with *Pantoea eucalypti* 299R [86]. This has also been observed in conditions where *B. cenocepacia* is individually non-motile (and does not produce surfactant) but uses *P. aeruginosa* rhamnolipids for motility [87, 88]. Further, flagella-deficient *P. aeruginosa* that produces rhamnolipids is incapable of swarming but can co-swarm with *B. cenocepacia* that is using the secreted surfactant [88], providing benefits for each species. Despite this display of hitchhiking on motile *B. cenocepacia*, in our study we did not observe cooperation between surfing *P. aeruginosa* and flagellar mutants (Fig. 2C), demonstrating that intraspecies hitchhiking is unlikely to contribute to this form of *P. aeruginosa* motility. However, the species providing the surfactants may benefit from the interaction if able to hitchhike on *P. aeruginosa*, although we did not see hitchhiking of *S. aureus* on the surfing *P. aeruginosa* population (Fig. 2C).

Biosurfactants not only reduce surface tension, but their properties like charge and toxicity can contribute to their roles as antimicrobials, inhibitors of biofilm formation, and emulsifiers [89]. For example, different classes of *P. aeruginosa* secreted surfactants mono- and di-rhamnolipids and their precursor 3-(3-hydroxyalkanoyloxy) alkanoic acids (HAAs) have opposing roles in coordinating tendril formation and motility during swarming [90]. In our experiments, we saw that in contrast to non-ionic Triton X-100 and anionic SDS, addition of the cationic detergent CTAB inhibited motility (Fig. 5A) [91], suggesting that charge could impact swarming and surfing. Taken together, we posit that biophysical characteristics of surfactants allow emergent motility, but their exact chemical attributes may alter the specifics of surfing.

Along with physical factors, nutrients such as carbon and nitrogen sources as well as iron availability also affect *P. aeruginosa* swarming [34, 41, 92, 93]. Transcriptional analysis of surfers compared to swarming or nonmotile cells, respectively, did not show changes in motility related genes. In conjunction with the observation that known major transcriptional regulators of motility are not required for surfing (Supp. Fig. 5 and 6), this suggests that the transition to surfing may not be regulated at the transcriptional level and may possibly be due to sensing of altered surface properties directly by the flagella [77].

There was an enrichment in heme biosynthesis and T3SS pathways in the downregulated genes in surfing cells. Further, on hard agar, transcripts of the phage φCTX were upregulated, and visible plaques were present especially in Las-deficient cells undergoing surfing on both semi-solid and hard agar (Supp. Fig. 5A and B). The T3SS is known to be regulated by a complex network involving both cyclic AMP and cyclic di-GMP signaling [63], where it is thought to be induced by low Ca^2+^ and host cell contact, and downregulated in a number of stress conditions, including DNA damage [61, 62]. DNA damage and other stresses also trigger prophage induction [64], suggesting that *P. aeruginosa* undergoing emergent motility may be sensing cellular stress. Heme biosynthesis genes and neighboring genes involved in nitrite reduction are induced by the anaerobic transcriptional regulator Dnr in conditions of low oxygen, as part of the nitrate respiration pathway [65]. Downregulation of these genes during surfing compared to swarming or non-motile cells suggests that swarming and non-motile cells are likely transitioning to anaerobic metabolism while surfers may still be engaging in oxidative respiration.

Some of the transcriptional responses seen in surfing cells are likely due to sensing unrelated *S. aureus* exoproducts. The upregulation of genes involved in TCA uptake and acetoin catabolism when surfing was also observed in our previous study in planktonic *P. aeruginosa* cells, where we determined the upregulation is a specific response to *S. aureus* secreted citrate and acetoin, respectively [57]. Thus, these staphylococcal intermediate metabolites are sensed by *P. aeruginosa* irrespective of the mode of growth and motility. Citrate and acetoin are likely not sufficient to induce surfing since the Agr-deficient *S. aureus* supernatant has similar levels of these nutrients as the WT [57], and still did not enable motility (Supp. Fig. 11A and D), but it is unclear how these or other nutrients may affect *P. aeruginosa* surfing. Additionally, viscosity, surface hardness, and osmolarity have been previously associated with altering *P. aeruginosa* motility [78, 91]. We observed surfing on both semi-solid and hard agar, a condition in which *P. aeruginosa* is nonmotile, however the effect of other physical parameters on surfing are yet to be characterized. While a diverse class of surfactants all transition *P. aeruginosa* to surfing surface motility, it is likely that the combination of physical and chemical cues from the host, neighboring microbial species, and the environment influence this motility and determine the resulting niche expansion and fitness changes in natural habitats.

## MATERIALS AND METHODS

### Bacterial strains and growth conditions.

Bacterial strains used in this study are listed in Supp. Table 6. *P. aeruginosa* UCBPP-PA14 [94], *S. aureus* JE2 [95], their derivatives, and other species were grown in a modified M63 medium [57] containing 1× M63 salts (13.6 g·L^−1^ KH_2_PO_4_, 2 g·L^−1^ (NH_4_)_2_SO_4_, 0.8 μM ferric citrate, 1 mM MgSO_4_; pH adjusted to 7.0 with KOH) supplemented with 0.3% glucose, 1× ACGU solution (Teknova), 1× supplement EZ (Teknova), 0.1 ng·L^−1^ biotin, and 2 ng·L^−1^ nicotinamide, at 37°C, with shaking at 300 rpm. *V. cholerae* was grown in M63 with 2% NaCl added. *S. aureus* and *P. aeruginosa* clinical isolates from the Cystic Fibrosis Foundation Isolate Core were selected at random from four different patients. *P. aeruginosa* and *S. aureus* transposon mutants were sourced from the PA14NR Set [96] and the Nebraska Transposon Mutant Library (NTML) [95], respectively, and confirmed by PCR using primers flanking the transposon insertion listed in Supp. Table 7. For cloning and mutant construction, strains were cultured in Luria Bertani (Miller) broth or on agar plates with 15 g·L^−1^ agar supplemented with 50 μg·mL^−1^ gentamicin and/or 25 μg·mL^−1^ irgasan as needed for selection.

### Preparation of cell-free supernatant.

Overnight cultures of bacterial strains were diluted to OD_600_ of 0.05 in fresh media and grown for 24 h in flasks before harvesting at 4000 rpm for 20 minutes. The supernatants were filter-sterilized with a Steriflip with a 0.2 μm polyethersulfone filter (MilliporeSigma). Supernatants were stored at −30°C until use.

### Agar plate motility assays.

LB agar plates (for twitch plates) or M63 agar plates (for twitch, swim, swarm, and hard agar plates) containing the specified percentage of agar (twitch: 1.5%; swim: 0.3%; swarm: 0.5%; hard: 1.5%) and 25% (v/v) media salts, media salts with the indicated additives, or the indicated species’ supernatant were prepared the day of the assay. For the twitch assays, M63 plates were used for experiments shown in Supp. Fig. 1B, and LB plates for all the other experiments. Freshly poured plates were allowed to rest at room temperature for 4 h before inoculation with the specified bacterial species. Plates were inoculated as follows: twitch, *P. aeruginosa* colony stabbed through the agar to the bottom of the plate; swim, *P. aeruginosa* overnight culture stabbed halfway through the agar; swarm and hard agar, 2 μL of *P. aeruginosa* overnight culture or mixed culture (equal volumes of both strains being mixed) was spotted on the center of the plate. Plates were then incubated for 24 h at 37°C in a single layer of petri dishes in a Mini Low Temperature Incubator (Fisherbrand) or for the twitch plates, for 48 h at 30°C. When noted, Type II mucin from porcine stomach (Sigma Aldrich), Triton X-100 (Fisher), sodium dodecyl sulfate (SDS) (Fisher BioReagents), hexadecyltrimethylammonium bromide (CTAB) (Sigma Aldrich), surfactin from *B. subtilis* (Sigma Aldrich), rhamnolipids from *P. aeruginosa* (Sigma Aldrich), Mannosylerythritol Lipid A (MEL-A) from *Pseudozyma* yeasts (Cayman), lactonic sophorolipid (Sigma Aldrich), LL-37 (Peptide Sciences), 1,2-Dipalmitoyl-sn-glycero-3-phosphocholine (DPPC) (Sigma Aldrich), and saponin from *Quillaja* bark (Sigma Aldrich) were added at the indicated concentrations to 25% media salts. After incubation, all plates were imaged either using a Chemidoc (Bio-Rad) on the white tray using the Coomassie blue setting or the black tray using the Cy2 (for GFP) or Cy3 (for dsRed or mKO) settings with optimal exposure, or for the twitch plates, using an Epson Perfection V700 Photo scanner with bottom illumination. All experiments were repeated three times.

### Construction of *P. aeruginosa* mutants.

Bacterial strains and plasmids are listed in Supp. Table 6. For mutant construction, homologous downstream and upstream arms of genes of interest were amplified using the primers listed in Supp. Table 7. The amplified fragments were cloned into pDONRPEX18Gm *attP* sites using the Gateway BP Clonase II Enzyme mix (ThermoFisher). Plasmids were transformed into *E. coli* S17–1 λ-pir and confirmed by sequencing prior to conjugation. Conjugants were streaked onto LB plates (without NaCl) + 10% sucrose, and then tested for gentamicin resistance. Gentamicin-sensitive strains were tested for the deletion by PCR and sequencing.

### RNA extraction and library preparation.

*P. aeruginosa* was scraped after 17 h from 1.5% or 0.5% agar M63 plates containing 25% (v/v) salts or *S. aureus* cell-free supernatant, resuspended in 2 mL salts mixed with 4 mL of RNAprotect Bacteria Reagent (Qiagen), and then incubated for 5 min at room temperature for stabilization before centrifugation at 4000 rpm for 10 min. Supernatants were completely removed before storage of the pellets at −80°C. RNA was extracted using the Total RNA Purification Plus Kit (Norgen) according to the manufacturer’s instructions for Gram-negative bacteria. The extracted RNA was subjected to an additional genomic DNA removal by DNase I treatment in solution using the TURBO DNA-free Kit (Invitrogen) and checked by PCR for the absence of contaminating DNA. Integrity of the RNA preparation was confirmed by running on an agarose gel and observing intact rRNA bands. Next, rRNA was removed using the *P. aeruginosa* riboPOOLs rRNA Depletion Kit (siTOOLs Biotech) followed by library preparation with the NEBNext Ultra II Directional RNA Library Prep Kit for Illumina (New England Biolabs). The sequencing was performed at the Center for Cancer Research (CCR) Genomics Core Facility. Two biological replicates were collected.

### RNA-seq analysis.

The sequencing files were processed with Cutadapt [97] and Trimmomatic [98]. Alignment to the *P. aeruginosa* UCBPP-PA14 genome (NCBI) and pairwise comparisons were made using Rockhopper (Wellesley College) [99, 100]. Gene expression was set at a minimum baseline value of 4 and *p* values of 0 were changed to the lowest values recorded within a dataset. Ribosomal RNAs and predicted RNAs were removed from each dataset. Upregulated and downregulated genes were based on transcripts that had *p* < 0.05 and log_2_ fold change ≥ 1 or ≤ −1. The location of prophages in the genome of *P. aeruginosa* UCBPP-PA14 NC_008463.1 was determined using PHASTER [101, 102]. Venn diagrams were generated using matplotlib_venn package with venn3 using Python [103].

### Gene ontology (GO) enrichment analysis.

For *P. aeruginosa* PA14 pathway analysis, the open reading frame designations for the corresponding PAO1 orthologs of *P. aeruginosa* UCBPP-PA14 genes were obtained using the *Pseudomonas* Genome Database (www.pseudomonas.com/rbbh/pairs) [104]. The list of designations from the RNA-seq analysis were analyzed at the Gene Ontology Resource (www.geneontology.org) by the PANTHER Overrepresentation Test (released 20221013 and 20231017, as noted) (Annotation Version and Release Date: GO Ontology database DOI: 10.5281/zenodo.7942786 Released 2023-05-10) for enriched biological processes by Fisher’s exact test with Bonferroni correction. All genes identified in the GO enrichment categories ‘heme biosynthesis’ and ‘T3SS’ as well as genes in the same or related neighboring operons, or known to be co-regulated, that were downregulated in both semi-solid and hard agar conditions, are shown in the heatmap in Fig. 3D.

## Supplementary Material

Supplement 1

Supplement 2

Supplement 3
