# Supplementary material for: Interspecies secreted surfactants induce emergent motility in Pseudomonas aeruginosa
Source: bioRxiv. 2024 Jan 4:2024.01.03.573969. Preprint. [Version 1] doi: 10.1101/2024.01.03.573969 (PMC10802355; doi:10.1101/2024.01.03.573969)
Supplement: Supplementary file 1 [file media-1.pdf]

SUPPLEMENTAL MATERIAL

TITLE

Interspecies secreted surfactants induce emergent motility in *Pseudomonas aeruginosa*

AUTHORS

Delayna L. Warrell[a*], Tiffany M. Zarrella[a,b,c#*], Christopher Machalek[a], Anupama Khare[a#]

[a]Laboratory of Molecular Biology, Center for Cancer Research, National Cancer Institute, National Institutes of Health, Bethesda, MD, USA

[b]Postdoctoral Research Associate Training Program, National Institute of General Medical Sciences, National Institutes of Health, Bethesda, MD, USA

[c]Current address: Department of Biology, Georgetown University, Washington, DC, USA

Running head: Surfactants induce motility in *P. aeruginosa*

[#]Address correspondence to Tiffany M. Zarrella, tiffany.zarrella@georgetown.edu, or Anupama Khare, anupama.khare@nih.gov

*Delayna L. Warrell and Tiffany M. Zarrella contributed equally to this work. Author order was determined alphabetically.

Key words: *Pseudomonas aeruginosa*, *Staphylococcus aureus*, surfactants, motility, polymicrobial interactions, phenol-soluble modulins

The authors declare no conflict of interest.

SUPPLEMENTAL FIGURES AND FIGURE LEGENDS

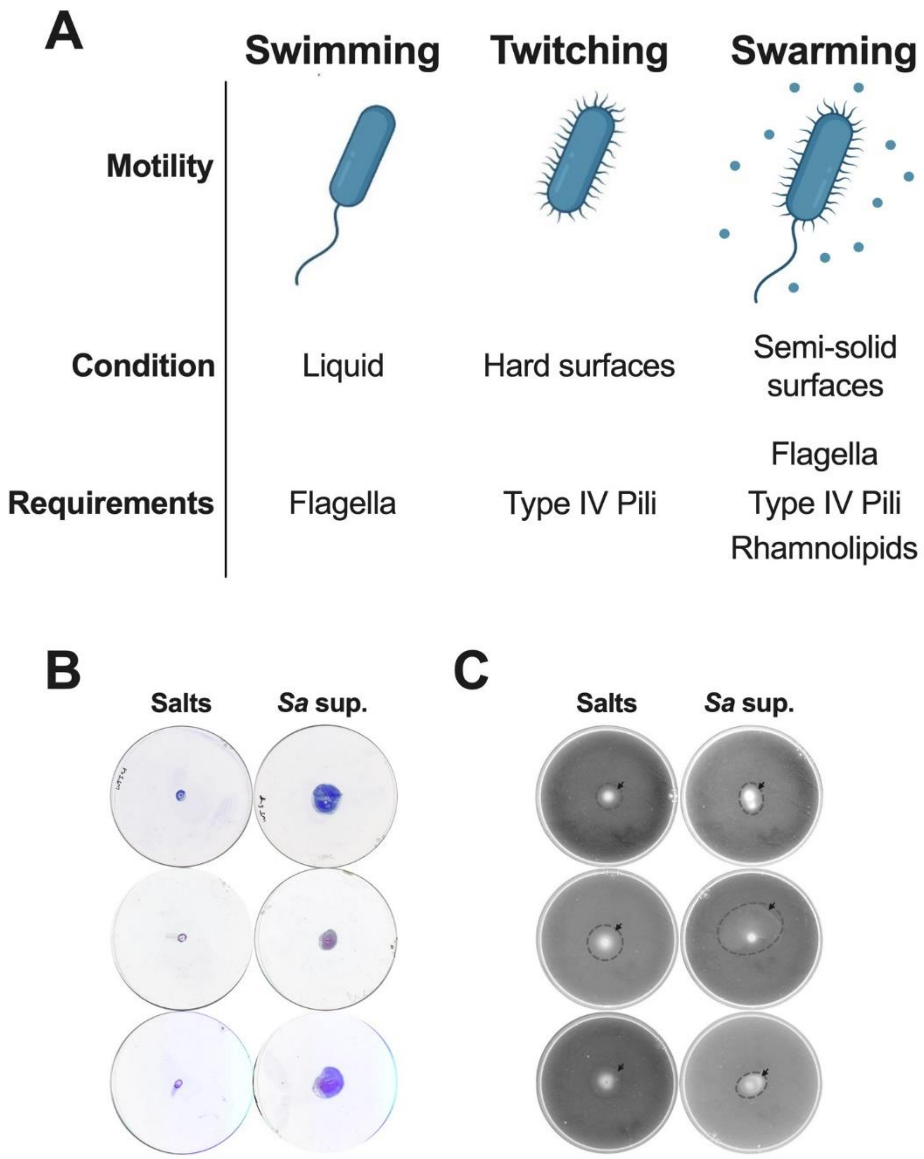

Supplemental Figure 1. *S. aureus* secreted products alter *P. aeruginosa* twitching and swimming motility. (A) (Left to right) *P. aeruginosa* swimming motility occurs in liquid conditions and requires flagella (1), twitching motility occurs on hard surfaces and requires type IV pili (2), and swarming motility occurs on semi-solid surfaces and requires flagella, type IV pili, and rhamnolipids (3, 4). **(B,C)** *P. aeruginosa* was inoculated on **(B)** twitch plates and **(C)** swim plates containing 25% media salts control or *S. aureus* supernatant. **(B)** Twitch plates were

incubated for 48 hours, visualized by crystal violet staining, and imaged. **(C)** Swim plates were imaged after 24 hours incubation. Arrows indicate the swim boundaries, and the dashed lines are drawn just outside the swim boundaries to depict the shape of the swim area. **(B,C)** Three independent replicates are shown.

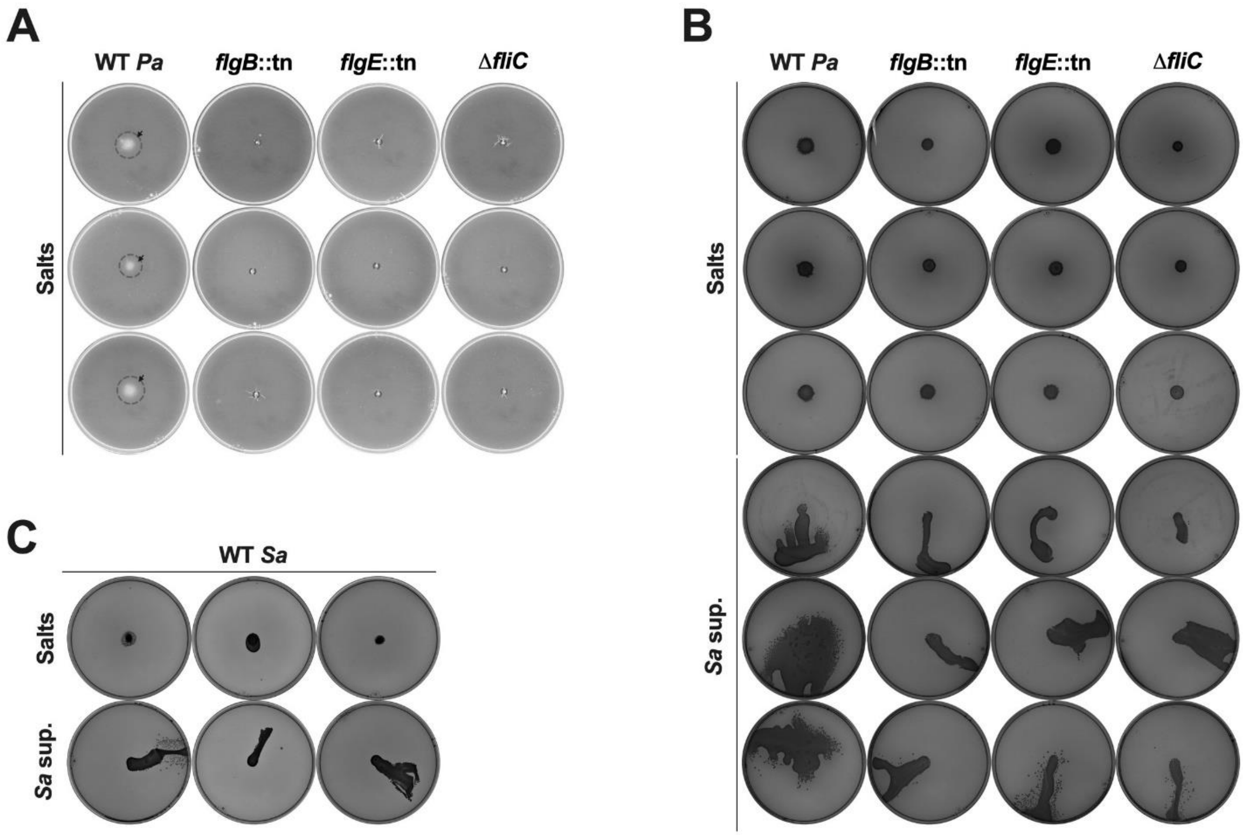

Supplemental Figure 2. ***P. aeruginosa*** **flagellar mutants do not exhibit emergent motility.** The indicated strains of **(A,B)** *P. aeruginosa* or **(C)** *S. aureus* were inoculated on **(A)** swim plates or **(B,C)** hard agar plates containing 25% media salts control or *S. aureus* supernatant, and motility was imaged after 24 hours incubation. **(A)** Arrows indicate the swim boundaries, and the dashed lines are drawn just outside the swim boundaries to depict the shape of the swim area. **(A-C)** Three independent replicates are shown.

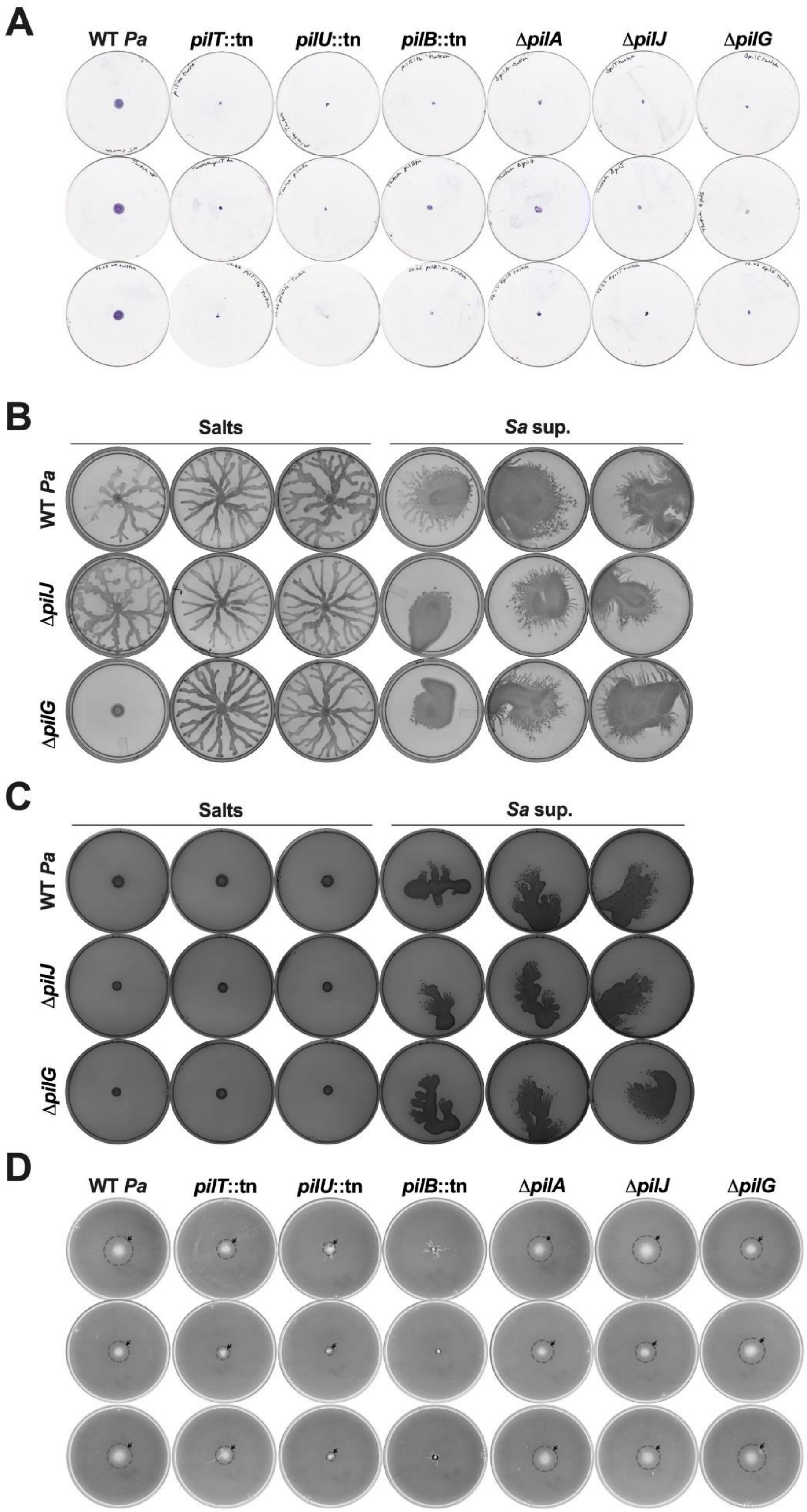
A
WT Pa
pilT::tn
pilU::tn
pilB::tn
ΔpilA
ΔpilJ
ΔpilG
B
Salts
Sa sup.
WT Pa
ΔpilJ
ΔpilG
C
Salts
Sa sup.
WT Pa
ΔpilJ
ΔpilG
D
WT Pa
pilT::tn
pilU::tn
pilB::tn
ΔpilA
ΔpilJ
ΔpilG

Supplemental Figure 3. *P. aeruginosa* pili mutants do not twitch but exhibit emergent motility. The indicated *P. aeruginosa* strains were inoculated on **(A)** twitch plates, **(B)** semi-solid agar plates, **(C)** hard agar plates, or **(D)** swim plates containing 25% media salts control or *S. aureus* supernatant. **(A)** Twitch plates composed of LB agar were inoculated with the indicated strain of *P. aeruginosa*, incubated for 48 hours, visualized by crystal violet staining, and imaged. **(B-D)** Plates were imaged after 24 hours incubation. **(D)** Arrows indicate the swim boundaries, and the dashed lines are drawn just outside the swim boundaries to depict the shape of the swim area. **(A-D)** Three independent replicates are shown.

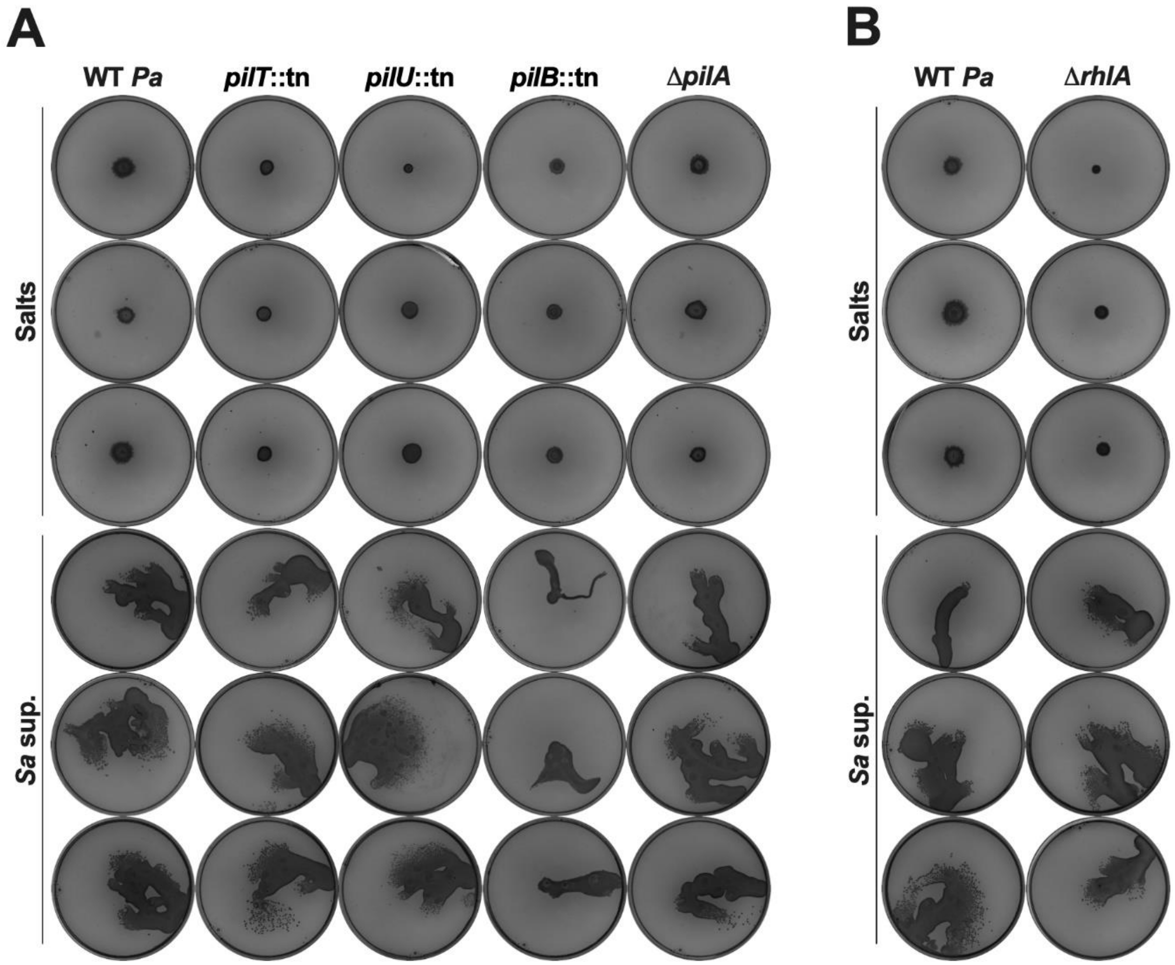

Supplemental Figure 4. Pili function and rhamnolipids are not required for emergent motility on hard agar. (A,B) The indicated *P. aeruginosa* strains were inoculated on hard agar plates containing 25% media salts control or *S. aureus* supernatant, and motility was imaged after 24 hours incubation. Three independent replicates are shown.

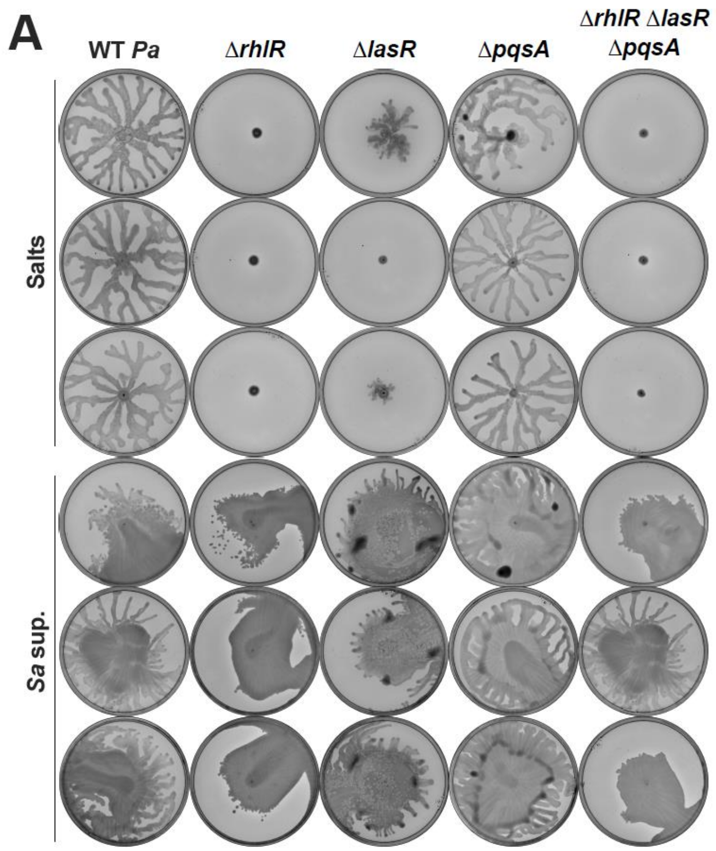
A
WT Pa
ΔrhlR
ΔlasR
ΔpqsA
ΔrhlR ΔlasR ΔpqsA
Salts
Sa sup.

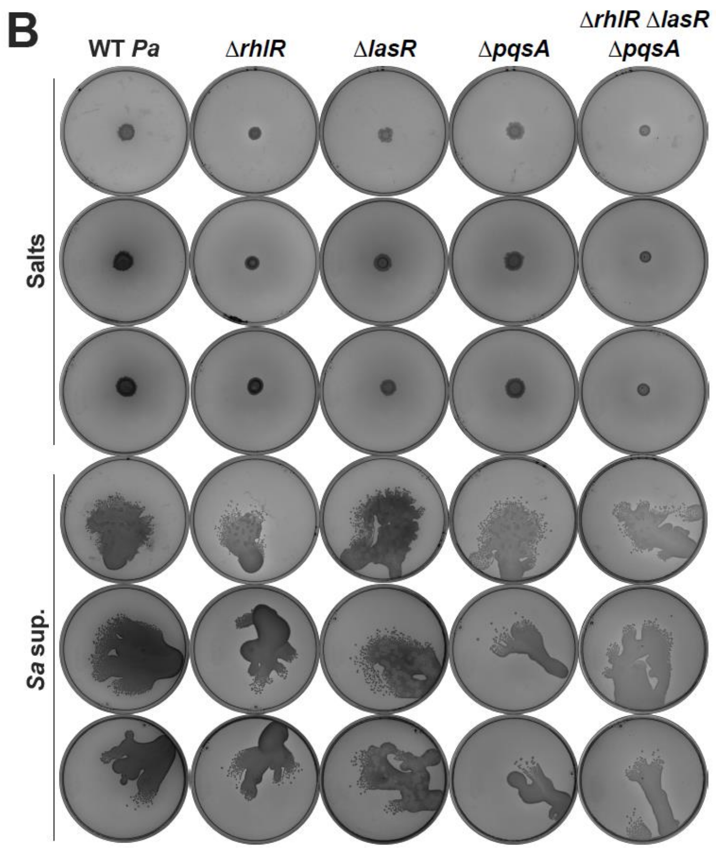
B
WT Pa
ΔrhlR
ΔlasR
ΔpqsA
ΔrhlR ΔlasR ΔpqsA
Salts
Sa sup.

Supplemental Figure 5. The *P. aeruginosa* quorum sensing systems are not required for emergent motility. The indicated *P. aeruginosa* strains were inoculated on **(A)** semi-solid or **(B)** hard agar plates containing 25% media salts control or *S. aureus* supernatant, and motility was imaged after 24 hours incubation. Three independent replicates are shown.

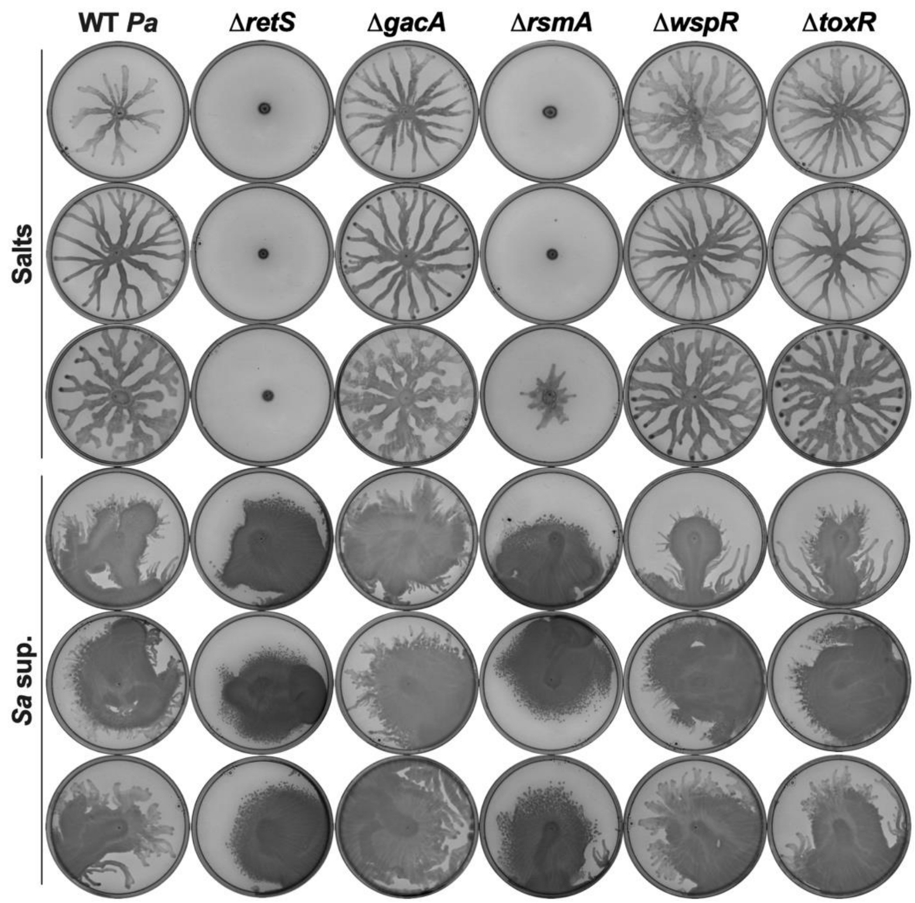

Supplemental Figure 6. Several *P. aeruginosa* regulators are not required for emergent motility. The indicated *P. aeruginosa* strains were inoculated on semi-solid agar plates containing 25% media salts control or *S. aureus* supernatant, and motility was imaged after 24 hours incubation. Three independent replicates are shown.

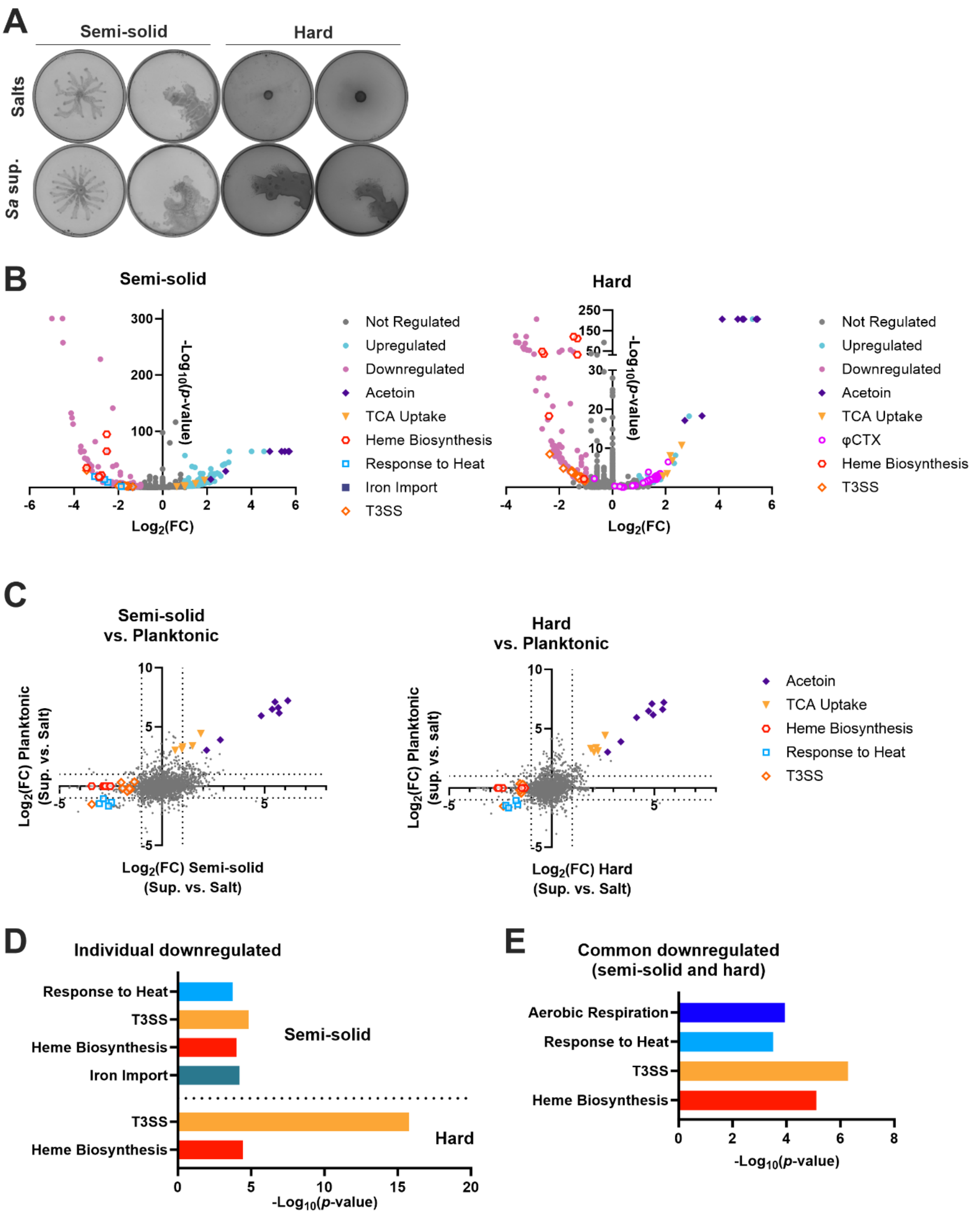

Supplemental Figure 7. *P. aeruginosa* differentially regulates genes when undergoing emergent motility vs. planktonic growth in the presence of *S. aureus* secreted products.

(A) WT *P. aeruginosa* cells were scraped from the leading edge on semi-solid and hard agar plates containing 25% media salts or *S. aureus* supernatant after 17 hours of incubation. Both independent replicates used for the RNA-seq are shown prior to scraping. **(B)** Volcano plots of -$\log_{10}$(*p*-value) vs. $\log_2$(fold-change) transcript levels after exposure to *S. aureus* supernatant compared to media salts control after 17 hours on **(left)** semi-solid or **(right)** hard agar. Genes shown as upregulated or downregulated have $p < 0.05$ and $\log_2$(fold-change) ≥ 1 or ≤ -1 respectively. **(C)** Scatter plots of $\log_2$(fold-change) transcript levels in *P. aeruginosa* after 17 hours on **(left)** semi-solid or **(right)** hard agar containing *S. aureus* supernatant compared to 2 hours exposure to *S. aureus* supernatant in planktonic culture (5). Genes shown as upregulated or downregulated have $p < 0.05$ and $\log_2$(fold-change) ≥ 1 or ≤ -1 respectively. **(D,E)** Gene Ontology (GO) enrichment (6-8) of *P. aeruginosa* genes downregulated after 17 hours incubation on **(D, top)** semi-solid and **(D, bottom)** hard agar plates or commonly downregulated on **(E)** both agar plates containing *S. aureus* supernatant. Nonredundant categories shown.

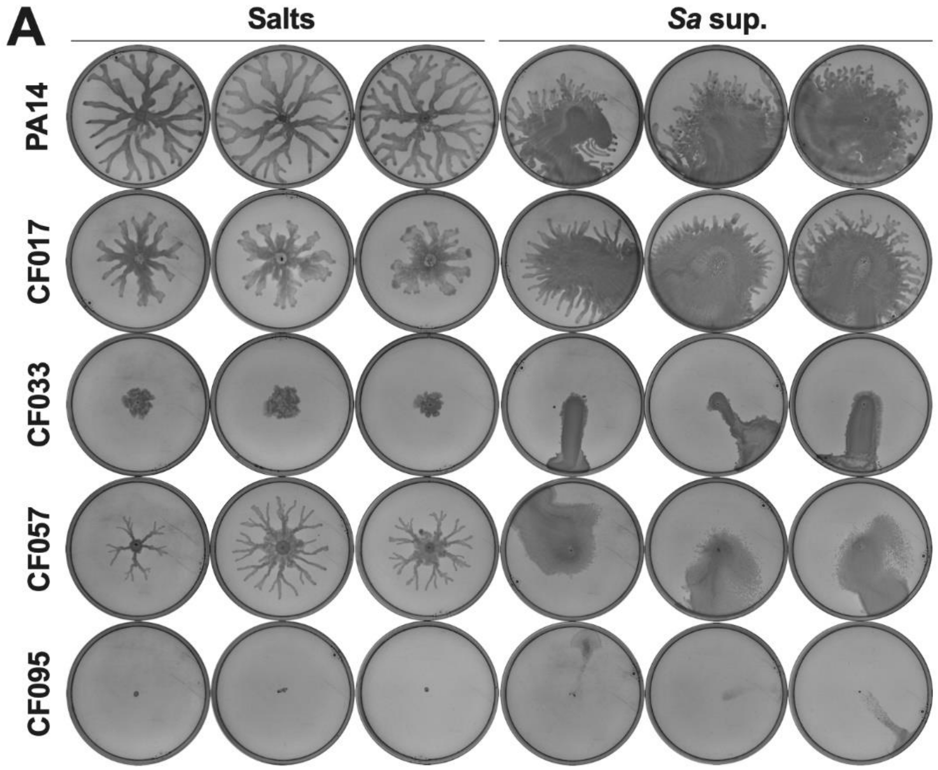
A
Salts
Sa sup.
PA14
CF017
CF033
CF057
CF095

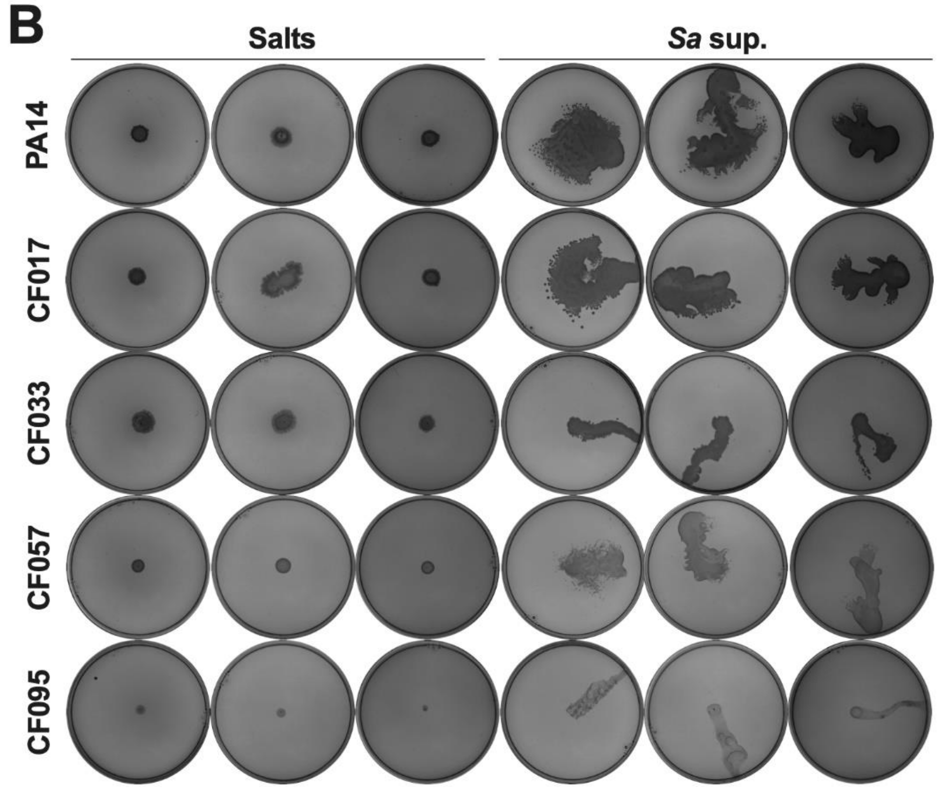
B
Salts
Sa sup.
PA14
CF017
CF033
CF057
CF095

Supplemental Figure 8. Clinical isolates of *P. aeruginosa* also exhibit emergent motility. The indicated *P. aeruginosa* strains were inoculated on **(A)** semi-solid or **(B)** hard agar plates containing 25% media salts control or *S. aureus* supernatant, and motility was imaged after 24 hours incubation.

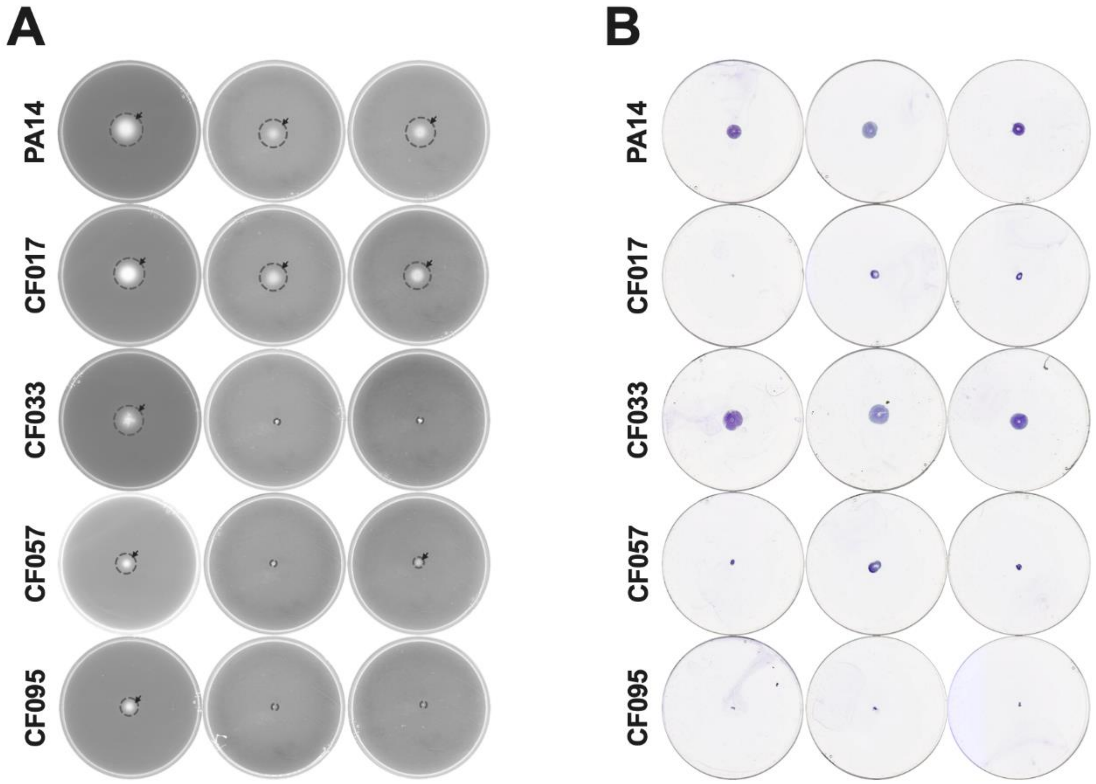

Supplemental Figure 9. *P. aeruginosa* clinical isolates show motility defects. The indicated *P. aeruginosa* strains were inoculated on **(A)** swim plates containing 25% media salts control or **(B)** twitch plates containing LB agar. **(A)** Swim plates were imaged after 24 hours incubation. Arrows indicate the swim boundaries, and the dashed lines are drawn just outside the swim boundaries to depict the shape of the swim area. **(B)** Twitch plates were incubated for 48 hours, visualized by crystal violet staining, and imaged. **(A,B)** Three independent replicates are shown.

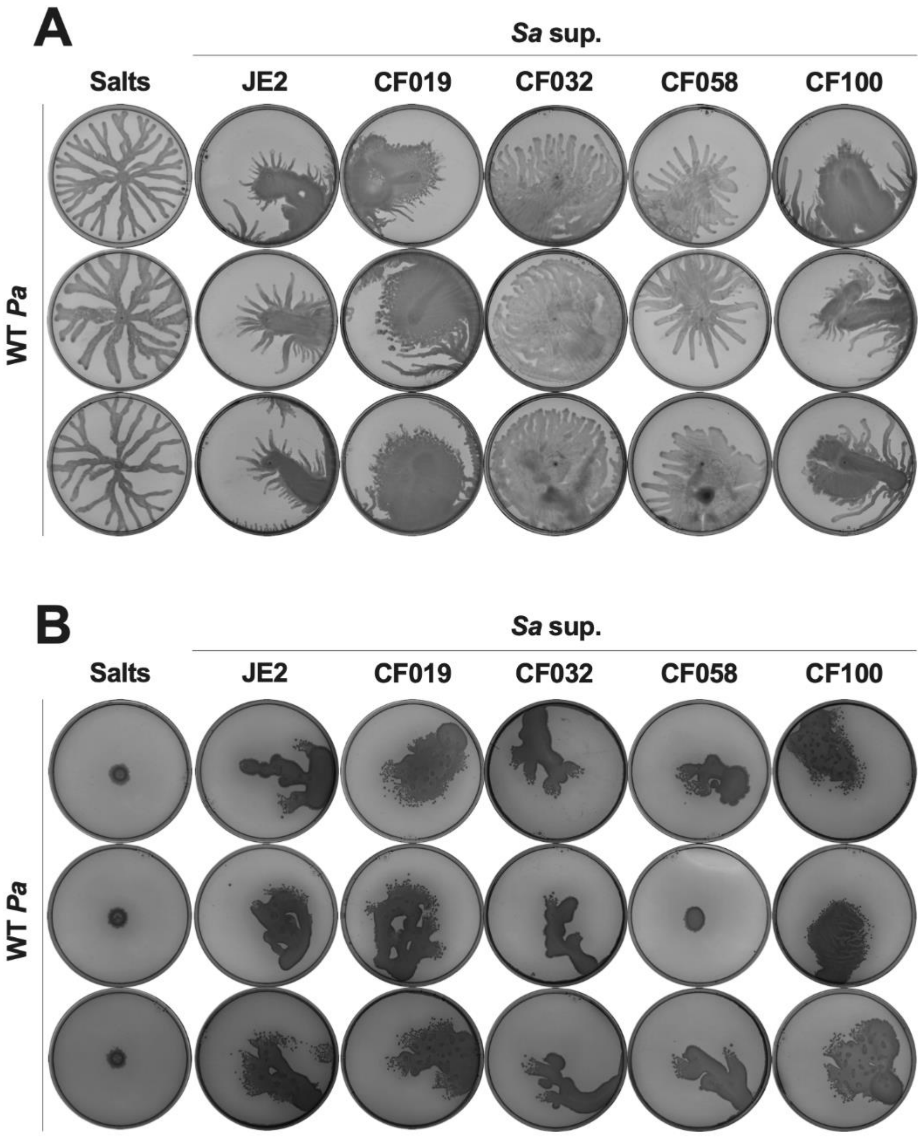

Supplemental Figure 10. *P. aeruginosa* exhibits emergent motility on supernatant from *S. aureus* clinical isolates. *P. aeruginosa* PA14 was inoculated on **(A)** semi-solid or **(B)** hard agar plates containing 25% media salts or 25% supernatant from the indicated *S. aureus* strains. Motility was imaged after 24 hours incubation. Three independent replicates are shown.

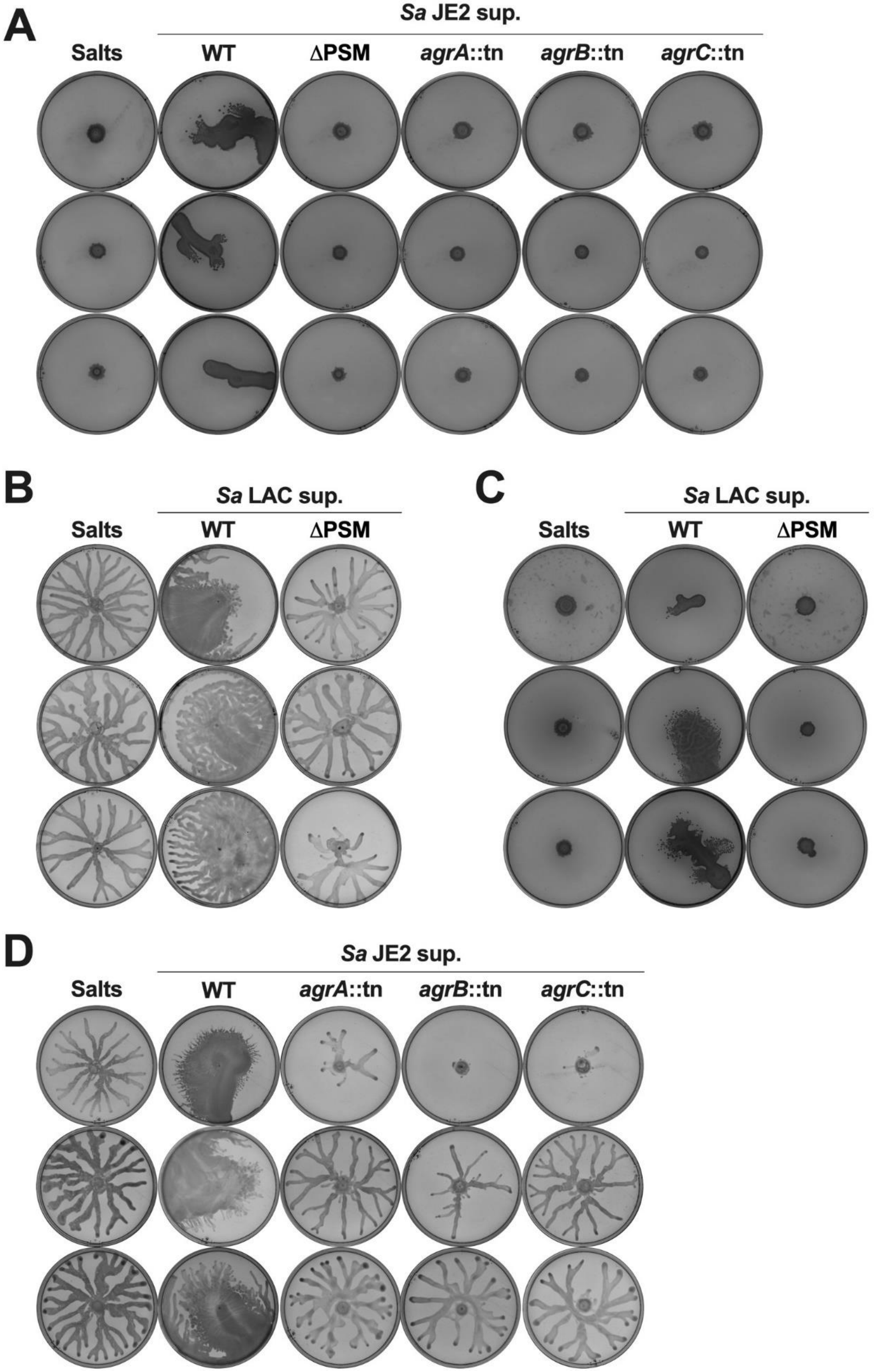
A
Sa JE2 sup.
Salts
WT
ΔPSM
agrA::tn
agrB::tn
agrC::tn
B
Sa LAC sup.
Salts
WT
ΔPSM
C
Sa LAC sup.
Salts
WT
ΔPSM
D
Sa JE2 sup.
Salts
WT
agrA::tn
agrB::tn
agrC::tn

Supplemental Figure 11. *P. aeruginosa* does not exhibit emergent motility on *S. aureus* supernatant lacking surfactant biosynthesis. *P. aeruginosa* was inoculated on **(A,C)** hard or **(B,D)** semi-solid agar plates containing 25% media salts or supernatant from the indicated *S. aureus* strains, and motility was imaged after 24 hours incubation. Three independent replicates are shown.

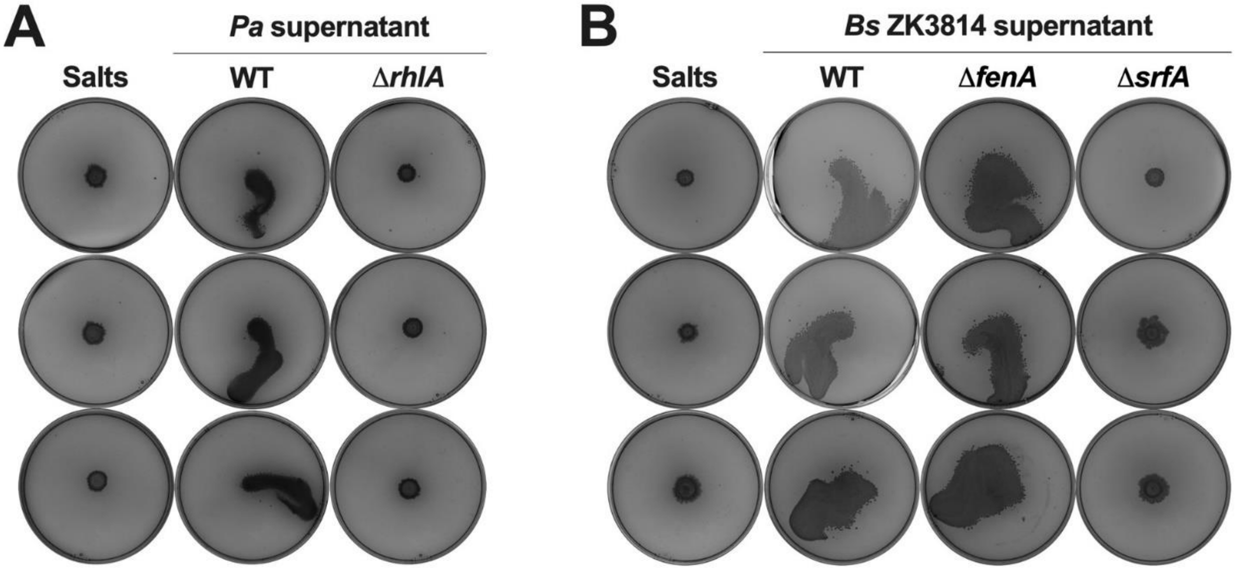

Supplemental Figure 12. Surfactant biosynthesis is required to induce emergent motility in *P. aeruginosa*. *P. aeruginosa* was inoculated on hard agar plates containing 25% salts or supernatant from the indicated strain of **(A)** *P. aeruginosa* or **(B)** *B. subtilis* ZK3814, and motility was imaged after 24 hours incubation. Three independent replicates are shown.

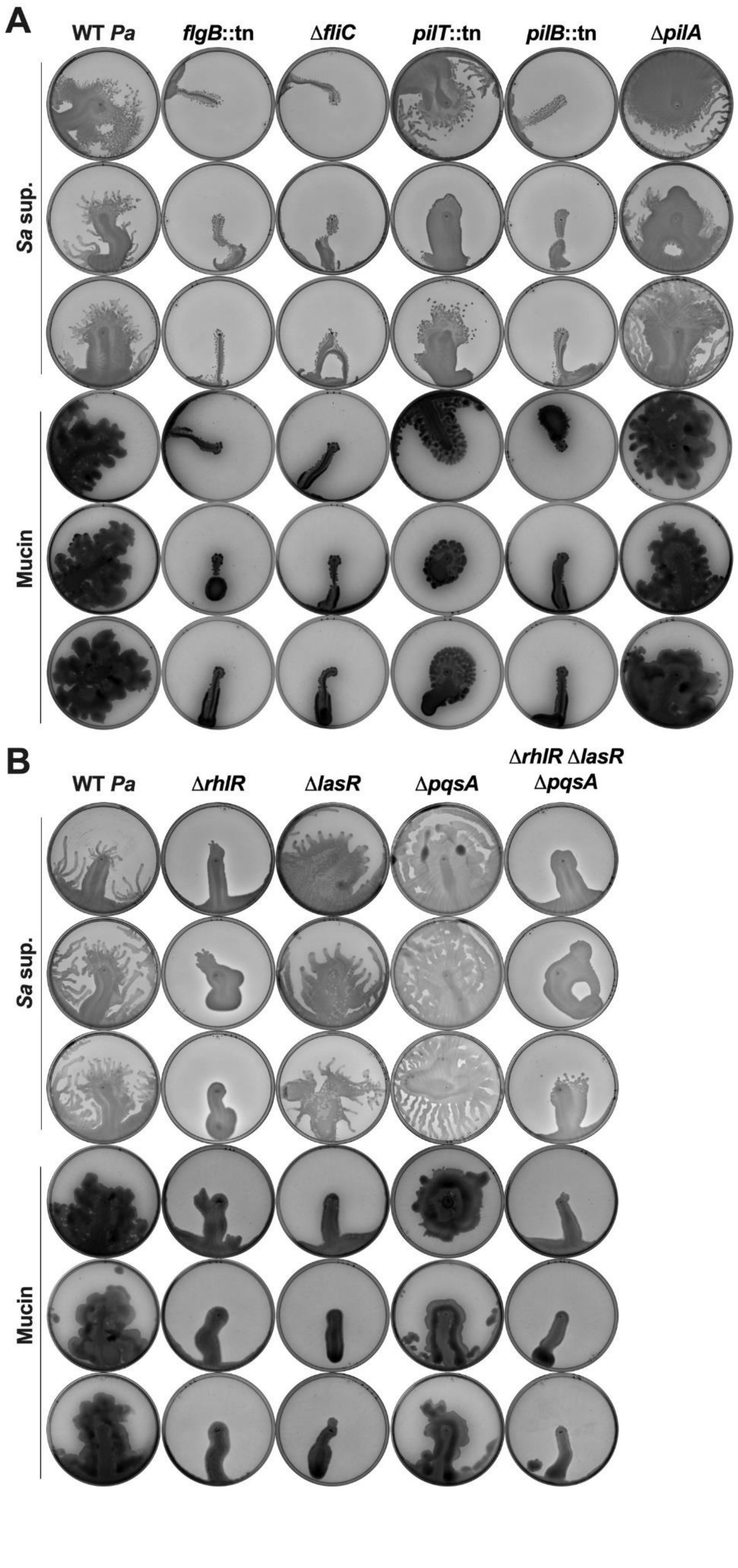
A
WT *Pa*
flgB::tn
Δ*fliC*
pilT::tn
pilB::tn
Δ*pilA*
Sa sup.
Mucin
B
WT *Pa*
Δ*rhlR*
Δ*lasR*
Δ*pqsA*
Δ*rhlR* Δ*lasR* Δ*pqsA*
Sa sup.
Mucin

Supplemental Figure 13. Quorum sensing and flagella, but not pili, are required for motility on mucin. (A,B) The indicated *P. aeruginosa* strains were inoculated on semi-solid agar plates containing 25% *S. aureus* supernatant or 25% media salts control with the addition of 0.4% mucin, and motility was imaged after 24 hours incubation. Three independent replicates are shown.

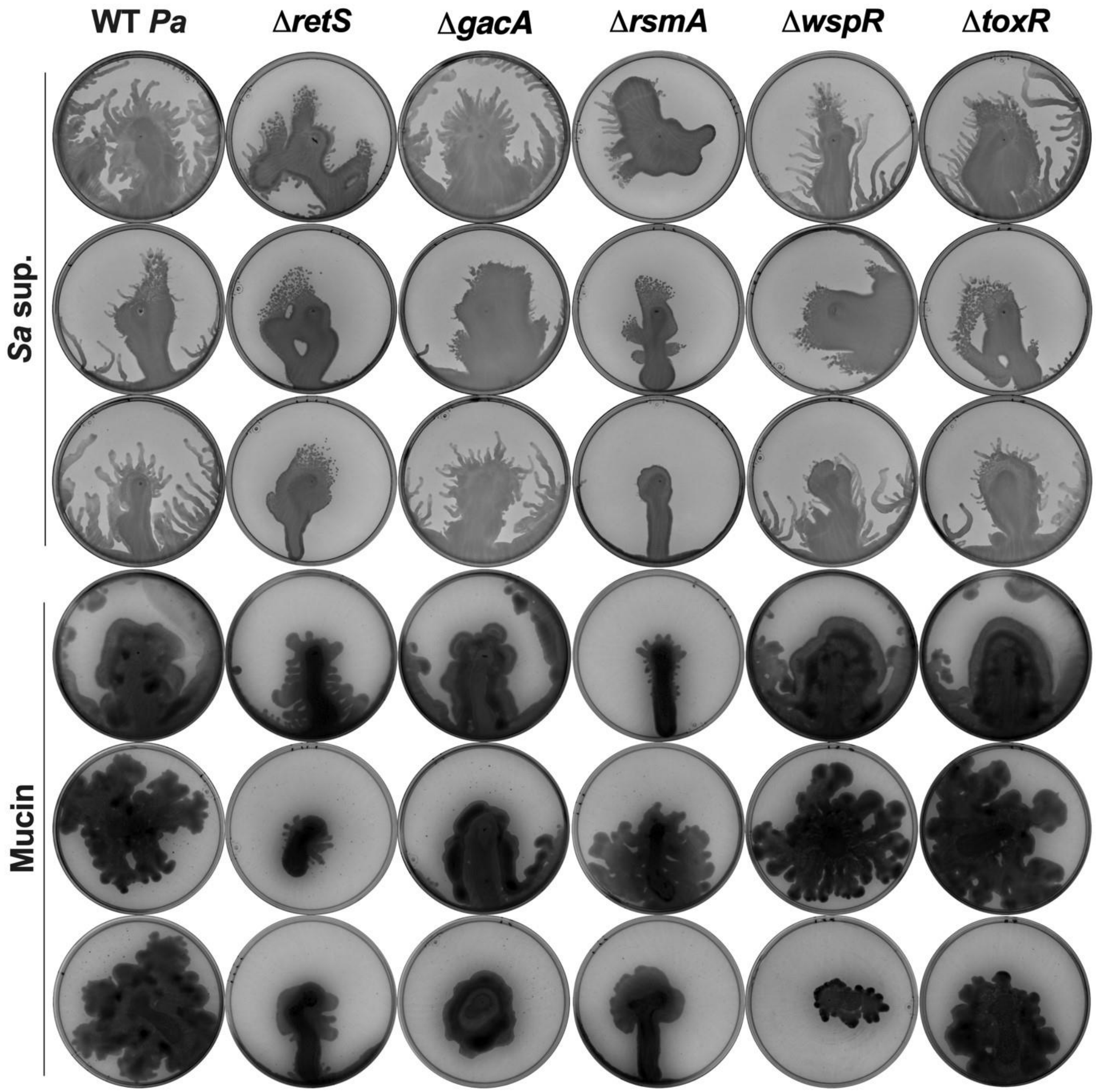

Supplemental Figure 14. Known *P. aeruginosa* motility regulatory systems are not required for movement on mucin. The indicated *P. aeruginosa* strains were inoculated on semi-solid agar plates containing 25% of *S. aureus* supernatant or 25% media salts control with the addition of 0.4% mucin, and images were taken after 24 hours incubation. Three independent replicates shown.

SUPPLEMENTAL TABLES

Supp. Table 1. Gene expression in *P. aeruginosa* from semi-solid or hard agar at 17 hours after exposure to *S. aureus* supernatant or media salts control determined from RNA-seq analysis. (Excel) Gene expression was analyzed by RNA-seq from RNA purified from two biological replicates of each treatment at 17 hours (semi-solid or hard agar with addition of *S. aureus* supernatant or media salts control).

Supp. Table 2. Differentially expressed genes between *P. aeruginosa* from semi-solid or hard agar plates after exposure to *S. aureus* supernatant or media salts control determined from RNA-seq analysis. (Excel) Gene expression was analyzed by RNA-seq from purified RNA from two biological replicates of each treatment after 17 hours (semi-solid or hard agar with addition of *S. aureus* supernatant or media salts control). Fold change indicates the mean expression of the supernatant-exposed *P. aeruginosa* over the control.

Supp. Table 3. Differentially expressed genes in common between *P. aeruginosa* from semi-solid and hard agar plates after exposure to *S. aureus* supernatant or media salts control determined from RNA-seq analysis. (Excel) Gene expression was analyzed by RNA-seq from purified RNA from two biological replicates of each treatment after 17 hours (semi-solid or hard agar with addition of *S. aureus* supernatant or media salts control). Genes commonly upregulated or downregulated from the semi-solid and hard agar conditions are listed. Fold change indicates the mean expression of the supernatant-exposed *P. aeruginosa* over the control.

Supp. Table 4. Differentially expressed genes in common between *P. aeruginosa* from semi-solid or hard agar and planktonic growth, or semi-solid and hard agar exclusively, after exposure to *S. aureus* supernatant or media salts control determined from RNA-seq

analysis. (Excel) Gene expression was analyzed by RNA-seq from purified RNA from two biological replicates of each treatment after 17 hours (semi-solid or hard agar and planktonic growth with addition of *S. aureus* supernatant or media salts control). Genes commonly upregulated or downregulated from the semi-solid or hard agar and planktonic growth conditions, or semi-solid and hard agar exclusively, are listed. Fold change indicates the mean expression of the supernatant-exposed *P. aeruginosa* over the control.

Supp. Table 5. Gene ontology (GO) enrichment of upregulated and downregulated genes after *P. aeruginosa* exposure to *S. aureus* exoproducts. (Excel) GO enrichment of *P. aeruginosa* genes differentially expressed on semi-solid and hard agar plates individually as well as in both, in common with planktonic cells, as well as exclusively in both motility conditions (but not in planktonic cells).

Supp. Table 6. Bacterial strains and plasmids used in this study.

Strain	Description[a,b]	Source
P. aeruginosa		
PA14	University of California Berkeley Plant Pathology (UCBPP)-PA14	(9)
33660	PA14 MAR2xT7 PA14_05180::tn (*pilT*); Gent^r	(10)
42562	PA14 MAR2xT7 PA14_50480::tn (*flgB*); Gent^r	(10)
48241	PA14 MAR2xT7 PA14_50450::tn (*flgE*); Gent^r	(10)
53607	PA14 MAR2xT7 PA14_05190::tn (*pilU*); Gent^r	(10)
56672	PA14 MAR2xT7 PA14_58750::tn (*pilB*); Gent^r	(10)
AK619	PA14 ΔPA14_51430 (*pqsA*) unmarked	(11)
CF017	AMT0194-10; co-isolated with CF019; age: 4.91 years	CFF Isolate Core
CF033	AMT0457-07; co-isolated with CF032; age: 4.28 years	CFF Isolate Core
CF057	AMT0461-12; co-isolated with CF058; age: 12.16 years	CFF Isolate Core
CF095	AMT0492-16; co-isolated with CF100; age: 13.52 years	CFF Isolate Core
SB049	PA14 expressing GFP; Gent^r	This study
SB092	PA14 ΔPA14_19100 (*rhlA*) unmarked	This study
SB387	PA14 ΔPA14_45960 (*lasR*) unmarked	This study
SB392	PA14 ΔPA14_19120 (*rhlR*) unmarked	This study
SB400	PA14 ΔPA14_05360 (*pilJ*) unmarked	This study
SB401	PA14 ΔPA14_05320 (*pilG*) unmarked	This study
SB402	PA14 ΔPA14_16500 (*wspR*) unmarked	This study
SB403	PA14 ΔPA14_55160 (*toxR*) unmarked	This study
SB426	PA14 ΔPA14_30650 (*gacA*) unmarked	This study
SB428	PA14 ΔPA14_58730 (*pilA*) unmarked	This study
SB431	PA14 ΔPA14_64230 (*retS*) unmarked	This study
SB446	PA14 ΔPA14_50290 (*fliC*) unmarked	This study
SB452	PA14 ΔPA14_45960 (*lasR*) ΔPA14_19120 (*rhlR*) ΔPA14_51430 (*pqsA*)	This study
SB454	PA14 ΔPA14_52570 (*rsmA*) unmarked	This study
SB485	SB446 expressing GFP	This study
SB489	PA14 expressing mKO	This study
S. aureus		
JE2	*Staphylococcus aureus* subsp. *aureus* USA300_FPR3757 (CA-MRSA)-JE2	(12)
NE95	JE2 SAUSA300_1989::tn (*agrB*); Em^r	(13)
NE873	JE2 SAUSA300_1991::tn (*agrC*); Em^r	(13)
NE1532	JE2 SAUSA300_1992::tn (*agrA*); Em^r	(13)
CF019	AMT0194-12; co-isolated with CF017; age: 4.91 years	CFF Isolate Core
CF032	AMT0457-03; co-isolated with CF033; age: 4.28 years	CFF Isolate Core
CF058	AMT0461-13; co-isolated with CF057; age: 12.16 years	CFF Isolate Core
CF100	AMT0492-21; co-isolated with CF095; age: 13.52 years	CFF Isolate Core
SB209	JE2 pKM16; Cm^r	This study
SB350	JE2 ΔPSM alpha1-4 delta (ATT)	D. Limoli; (14)
SB371	LAC	M. Otto
SB372	LAC ΔPSM alpha1-4 beta1-2 delta (ATT)	M. Otto; (15)

E. coli		
ccdB Survival 2 T1^R	*E. coli* strain used for maintenance of pDONR plasmid	Invitrogen
DH5α	*E. coli* strain used for cloning	NEB
IM08B	*E. coli* strain used for cloning	BEI Resources
S17-1 λ-pir	*E. coli* strain used for conjugation	(16)
AK111	MG1655 SB144	(17)
Other species		
SB145	*Bacillus subtilis* PY79	K. Ramamurthi
SB146	*Burkholderia cenocepacia*; ATCC 25608	S. Adhya
SB147	*Klebsiella pneumoniae* subsp. *pneumoniae* KPNIH1	S. Adhya
SB149	*Vibrio cholerae*	S. Adhya
SB373	*B. subtilis* ZK3814	M. Otto; (18)
SB374	ZK3814 Δ*fenA*	M. Otto; (18)
SB375	ZK3814 Δ*srfA*	M. Otto; (18)
SB468	*B. cenocepacia*; ATCC BAA-245	S. Adhya
**Plasmids**		
Gene deletion		
pDONRPEX18Gm	Shuttle vector with *attP* sites and *ccdB*; Cm^r Gent^r	(19)
pAK611	pEX18ApGW: ΔPA14_51430 (*pqsA*); Ap^r Gent^r	(11)
pSB445	pDONRPEX18Gm: ΔPA14_50290 (*fliC*); Gent^r	This study
pSB491	pDONRPEX18Gm: ΔPA14_19100 (*rhlA*); Gent^r	This study
pSB388	pDONRPEX18Gm: ΔPA14_45960 (*lasR*); Gent^r	This study
pSB391	pDONRPEX18Gm: ΔPA14_19120 (*rhlR*); Gent^r	This study
pSB396	pDONRPEX18Gm: ΔPA14_05360 (*pilJ*); Gent^r	This study
pSB397	pDONRPEX18Gm: ΔPA14_05320 (*pilG*); Gent^r	This study
pSB398	pDONRPEX18Gm: ΔPA14_16500 (*wspR*); Gent^r	This study
pSB399	pDONRPEX18Gm: ΔPA14_55160 (*toxR*); Gent^r	This study
pSB420	pDONRPEX18Gm: ΔPA14_64230 (*retS*); Gent^r	This study
pSB421	pDONRPEX18Gm: ΔPA14_30650 (*gacA*); Gent^r	This study
pSB422	pDONRPEX18Gm: ΔPA14_58730 (*pilA*); Gent^r	This study
pSB453	pDONRPEX18Gm: ΔPA14_52570 (*rsmA*); Gent^r	This study
Express fluorescent marker		
pKM16	Expressing DsRed from SarA-P1 promoter; Ap^r Cm^r	S. Brinsmade; (20)
pUC18T-mini-Tn7T-Gm-*gfpmut3*	Expressing GFP; Ap^r Gent^r	H. Schweizer; (21)
pUC18T-mini-Tn7T-Gm-*mKO*	Expressing mKO; Ap^r Gent^r	H. Schweizer; (21)

[a] Ap^{r}, ampicillin resistance (*E. coli*); Cm^{r}, chloramphenicol resistance (*E. coli*, *S. aureus*); Em^{r}, erythromycin resistance (*S. aureus*); Gent^{r}, gentamicin resistance (*P. aeruginosa*)

[b] CFF Isolate Core Samples are annotated as follows: AMT####-## (Patient ID-Isolate number)

Supp. Table 7. Primers used in this study*.

Primer	Sequence 5′→3′	Site^	Location*	Application
Pa003	ggacttatcagccaacctgtt	-	MAR2xT7 transposon	Verify transposon insertion
Pa061	ggggacaagtttgtacaaaaaagcaggctcaccatgtgaccctcgagttct	*attB1*	Up PA14_19100	Generate mutant
Pa062	ggttcaggcgtagccgatcatctcacacctcccaaaaa	-	Up and down PA14_19100	Generate mutant
Pa063	tttttgggaggtgtgagatgatcggctacgcctgaacc	-	Up and down PA14_19100	Generate mutant
Pa064	ggggaccactttgtacaagaaagctgggtacacgttgaacttggggtgta	*attB2*	Down PA14_19100	Generate mutant
Pa065#	aaatcggacaagtggattcg	-	Up PA14_19100	Sanger sequencing
Pa066#	atcgagaaagcgttgcagtt	-	Down PA14_19100	Sanger sequencing
Pa108	tgaagaaccaggacccgatg		Up PA14_50450	Verify transposon insertion
Pa109	gctcatggagacgtacagca		Down PA14_50450	Verify transposon insertion
Pa229#	gctgttcgacggcagtatc	-	Up PA14_19120	Sanger sequencing
Pa230	ggggacaagtttgtacaaaaaagcaggctcaaactgcaacgctttctcgat	*attB1*	Up PA14_19120	Generate mutant
Pa231	tacgcttcagatgaggcccagcaaaaagcctccgtcattcct	-	Up and down PA14_19120	Generate mutant
Pa232#	aacggctgacgacctcac	-	Down PA14_19120	Sanger sequencing
Pa233	aggaatgacggaggctttttgctgggcctcatctgaagcgta	-	Up and down PA14_19120	Generate mutant
Pa234	ggggaccactttgtacaagaaagctgggtagtggtagcgcaacagcatct	*attB2*	Down PA14_19120	Generate mutant
Pa312	ggggacaagtttgtacaaaaaagcaggctcattctacctgctcaacagccg	*attB1*	Up PA14_45960	Generate mutant
Pa313	agaggcaagatcagagagtaataagaccgtcaaccaaggccatagc	-	Up and down PA14_45960	Generate mutant
Pa314	gctatggccttggttgacggtcttattactctctgatcttgcctct	-	Up and down PA14_45960	Generate mutant
Pa315	ggggaccactttgtacaagaaagctgggtagaaacggctgagttcccaga	*attB2*	Down PA14_45960	Generate mutant
Pa316#	tcaacatggtcacctccagc	-	Up PA14_45960	Sanger sequencing
Pa317#	tccagcgtacagtcggaaaag	-	Down PA14_45960	Sanger sequencing

Pa339	ggggacaagtttgtacaaaaaagcaggctcaggacgaagagaccctgctga	*attB1*	Up PA14_05360	Generate mutant
Pa340	cctatgctcaggcctgctctcttcatatttggcccccgc	-	Up and down PA14_05360	Generate mutant
Pa341	gcgggggccaaatatgaagagagcaggcctgagcatagg	-	Up and down PA14_05360	Generate mutant
Pa342	ggggaccactttgtacaagaaagctgggtacaggtccagcacattcagcc	*attB2*	Down PA14_05360	Generate mutant
Pa343[#]	ccaggtactcaaggccgaga	-	Up PA14_05360	Sanger sequencing
Pa344[#]	tgcctgagtaccccttacgg	-	Down PA14_05360	Sanger sequencing
Pa345	ggggacaagtttgtacaaaaaagcaggctcagggtgagggtctcgaggatc	*attB1*	Up PA14_05320	Generate mutant
Pa346	gcggccggatatcaggaaacctgttccatgttcgccctata	-	Up and down PA14_05320	Generate mutant
Pa347	tatagggcgaacatggaacaggtttcctgatatccggccgc	-	Up and down PA14_05320	Generate mutant
Pa348	ggggaccactttgtacaagaaagctgggtatgcatgccgaacacctcatc	*attB2*	Down PA14_05320	Generate mutant
Pa349[#]	cgccgtcgatcatcaggatg	-	Up PA14_05320	Sanger sequencing
Pa350[#]	tcgaggaagccctggtgttg	-	Down PA14_05320	Sanger sequencing
Pa351	ggggacaagtttgtacaaaaaagcaggctcacgataaccgtggcaaggtcc	*attB1*	Up PA14_16500	Generate mutant
Pa352	gcggcaccggctgttcgtgcatgtttctctccggga	-	Up and down PA14_16500	Generate mutant
Pa353	tcccggagagaaacatgcacgaacagccggtgccgc	-	Up and down PA14_16500	Generate mutant
Pa354	ggggaccactttgtacaagaaagctgggtacgtactcgcccttgatgtgc	*attB2*	Down PA14_16500	Generate mutant
Pa355[#]	tcatggcggaaagtccttgc	-	Up PA14_16500	Sanger sequencing
Pa356[#]	tcatcgcggtgtccttgttg	-	Down PA14_16500	Sanger sequencing
Pa357	ggggacaagtttgtacaaaaaagcaggctcaggagagctggacctgatcgt	*attB1*	Up PA14_55160	Generate mutant
Pa358	atcggcgttcagcaggctgtcgcagtcataagtgatggc	-	Up and down PA14_55160	Generate mutant
Pa359	gccatcacttatgactgcgacagcctgctgaacgccgat	-	Up and down PA14_55160	Generate mutant
Pa360	ggggaccactttgtacaagaaagctgggtacgggcccgagaaaaatcctc	*attB2*	Down PA14_55160	Generate mutant
Pa361[#]	gaaccgcctgctggtcac	-	Up PA14_55160	Sanger sequencing
Pa362[#]	tgtgtcgttcttgtcactcgt	-	Down PA14_55160	Sanger sequencing

Pa367	tcgaccagcaggacatcaac	-	Up PA14_50480	Verify transposon insertion
Pa368	atgctcatgctctactccgc	-	Down PA14_50480	Verify transposon insertion
Pa369	agggtagagtcagccggaat	-	Up PA14_58750	Verify transposon insertion
Pa370	gccttcaccagcatagggtt	-	Down PA14_58750	Verify transposon insertion
Pa371	caatcggtgatctcgcagacc	-	Up PA14_05190	Verify transposon insertion
Pa372	aggaaatccagttggccgag	-	Down PA14_05190	Verify transposon insertion
Pa373	actggatggggccgatgaa	-	Up PA14_05180	Verify transposon insertion
Pa374	ataccatgcccaccaggttg	-	Down PA14_05180	Verify transposon insertion
Pa381	ggggacaagtttgtacaaaaaagcaggctcacgaagtgatctacccgacgg	*attB1*	Up PA14_64230	Generate mutant
Pa382	gtcgctgccctcaggagatccgaagccgtaccac	-	Up and down PA14_64230	Generate mutant
Pa383	gtggtacggcttcggatctcctgagggcagcgac	-	Up and down PA14_64230	Generate mutant
Pa384	ggggaccactttgtacaagaaagctgggtagatgaagatgtagcggccga	*attB2*	Down PA14_64230	Generate mutant
Pa385[#]	aggaggccagcttcatcgt	-	Up PA14_64230	Sanger sequencing
Pa386[#]	gcagacgaacagacccatca	-	Down PA14_64230	Sanger sequencing
Pa387	ggggacaagtttgtacaaaaaagcaggctcactgatcctgttccagcagca	*attB1*	Up PA14_30650	Generate mutant
Pa388	atctagctggcggcatcgac(aatcacgctgcacctcgtc)	-	Up and down PA14_30650	Generate mutant
Pa389	gacgaggtgcagcgtgatt(gtcgatgccgccagctagat)	-	Up and down PA14_30650	Generate mutant
Pa390	ggggaccactttgtacaagaaagctgggtattgcagcgcttgatctggta	*attB2*	Down PA14_30650	Generate mutant
Pa391[#]	ctgatcctgttccagcagca	-	Up PA14_30650	Sanger sequencing
Pa392[#]	cctgctccatgccgacg	-	Down PA14_30650	Sanger sequencing
Pa393	ggggacaagtttgtacaaaaaagcaggctcacaagtacaacgttccgctgc	*attB1*	Up PA14_52570	Generate mutant

Pa394	ttaatggtttggctcttgatctttcattcctttctcctcacgcgaat	-	Up and down PA14_52570	Generate mutant
Pa395	attcgcgtgaggagaaaggaatgaaagatcaagagccaaaccattaa	-	Up and down PA14_52570	Generate mutant
Pa396	ggggaccactttgtacaagaaagctgggtaaaactgctctaccgccttcc	*attB2*	Down PA14_52570	Generate mutant
Pa397[#]	ggcgtctacaccaccgatc	-	Up PA14_52570	Sanger sequencing
Pa398[#]	aaactgctctaccgccttcc	-	Down PA14_52570	Sanger sequencing
Pa399	ggggacaagtttgtacaaaaaagcaggctcactcgatgatgatgccgagct	*attB1*	Up PA14_58730	Generate mutant
Pa400	tcttttcagcattagcctattagcgctgagctttcatgtatctctccattg	-	Up and down PA14_58730	Generate mutant
Pa401	caatggagagatacatgaaagctcagcgctaataggctaatgctgaaaag a	-	Up and down PA14_58730	Generate mutant
Pa402	ggggaccactttgtacaagaaagctgggtaccaattgggtctgtagcggt	*attB2*	Down PA14_58730	Generate mutant
Pa403[#]	cgttcggagatatccaggcc	-	Up PA14_58730	Sanger sequencing
Pa404[#]	ctacatctccatcggcaccc	-	Down PA14_58730	Sanger sequencing
Pa405	ggggacaagtttgtacaaaaaagcaggctcatccctatgtgcgcgaatacc	*attB1*	Up PA14_50290	Generate mutant
Pa406	cagctggttggcctgggcaagggccatggtgatttcc	-	Up and down PA14_50290	Generate mutant
Pa407	ggaaatcaccatggcccttgcccaggccaaccagctg	-	Up and down PA14_50290	Generate mutant
Pa408	ggggaccactttgtacaagaaagctgggtaatcggtgtaatcggtcgagc	*attB2*	Down PA14_50290	Generate mutant
Pa409[#]	tccctatgtgcgcgaatacc	-	Up PA14_50290	Sanger sequencing
Pa410[#]	cgacggagatgttcagcgta	-	Down PA14_50290	Sanger sequencing
Sa046	ctcgattctattaacaaggg	-	"Upstream" *bursa aurealis* transposon	Verify transposon insertion
Sa047	gctttttctaaatgtttttaagtaaatca	-	"Buster" *bursa aurealis* transposon	Verify transposon insertion
Sa094	agaaaagcctatggaaattgccctc	-	Up SAUSA300_ 1992	Verify transposon insertion
Sa095	tcaccgatgcatagcagtgt	-	Down SAUSA300_ 1992	Verify transposon insertion

Sa096	gtataatgacagtgaggagagtggtg	-	Up SAUSA300_1989	Verify transposon insertion
Sa097	aggacgcgctatcaaacatt	-	Down SAUSA300_1989	Verify transposon insertion
Sa098	gagagtgtgatagtaggtggaattat	-	Up SAUSA300_1991	Verify transposon insertion
Sa099	gcgtggtatatcatcagcgc	-	Down SAUSA300_1991	Verify transposon insertion

* Up = upstream arm of gene; down = downstream arm of gene.

[#] Primers utilized for Sanger sequencing.

^ Site is underlined in primer sequence.

SUPPLEMENTAL REFERENCES

1. Ha DG, Kuchma SL, O'Toole GA. 2014. Plate-based assay for swimming motility in *Pseudomonas aeruginosa*. Methods Mol Biol 1149:59-65.

2. Burrows LL. 2012. *Pseudomonas aeruginosa* twitching motility: type IV pili in action. Annu Rev Microbiol 66:493-520.

3. Köhler T, Curty LK, Barja F, van Delden C, Pechère JC. 2000. Swarming of *Pseudomonas aeruginosa* is dependent on cell-to-cell signaling and requires flagella and pili. J Bacteriol 182:5990-6.

4. Caiazza NC, Shanks RM, O'Toole GA. 2005. Rhamnolipids modulate swarming motility patterns of *Pseudomonas aeruginosa*. J Bacteriol 187:7351-61.

5. Zarrella TM, Khare A. 2022. Systematic identification of molecular mediators of interspecies sensing in a community of two frequently coinfecting bacterial pathogens. PLoS Biol 20:e3001679.

6. Ashburner M, Ball CA, Blake JA, Botstein D, Butler H, Cherry JM, Davis AP, Dolinski K, Dwight SS, Eppig JT, Harris MA, Hill DP, Issel-Tarver L, Kasarskis A, Lewis S, Matese JC, Richardson JE, Ringwald M, Rubin GM, Sherlock G. 2000. Gene Ontology: tool for the unification of biology. The Gene Ontology Consortium. Nat Genet 25:25-9.

7. Mi H, Muruganujan A, Ebert D, Huang X, Thomas PD. 2019. PANTHER version 14: more genomes, a new PANTHER GO-slim and improvements in enrichment analysis tools. Nucleic Acids Res 47:D419-D426.

8. Gene Ontology C. 2021. The Gene Ontology resource: enriching a GOld mine. Nucleic Acids Res 49:D325-D334.

9. Rahme LG, Stevens EJ, Wolfort SF, Shao J, Tompkins RG, Ausubel FM. 1995. Common virulence factors for bacterial pathogenicity in plants and animals. Science 268:1899-902.

10. Liberati NT, Urbach JM, Miyata S, Lee DG, Drenkard E, Wu G, Villanueva J, Wei T, Ausubel FM. 2006. An ordered, nonredundant library of Pseudomonas aeruginosa strain PA14 transposon insertion mutants. Proc Natl Acad Sci U S A 103:2833-8.

11. Khare A, Tavazoie S. 2015. Multifactorial competition and resistance in a two-species bacterial system. PLoS Genet 11:e1005715.

12. Kennedy AD, Otto M, Braughton KR, Whitney AR, Chen L, Mathema B, Mediavilla JR, Byrne KA, Parkins LD, Tenover FC, Kreiswirth BN, Musser JM, DeLeo FR. 2008. Epidemic community-associated methicillin-resistant *Staphylococcus aureus*: recent clonal expansion and diversification. Proc Natl Acad Sci U S A 105:1327-32.

13. Fey PD, Endres JL, Yajjala VK, Widhelm TJ, Boissy RJ, Bose JL, Bayles KW. 2013. A genetic resource for rapid and comprehensive phenotype screening of nonessential *Staphylococcus aureus* genes. MBio 4:e00537-12.

14. Wang GZW, E. A.; Haas, A. L.; Sánchez Peña, A.; Kiedrowski, M. R.; Lomenick, B.; Chou, T.; Bomberger, J. M.; Tirrell, D. A.; Limoli, D. H. 2023. *Staphylococcal* secreted cytotoxins are competition sensing signals for *Pseudomonas aeruginosa*. bioRxiv doi:https://doi.org/10.1101/2023.01.29.526047.

15. Dastgheyb SS, Villaruz AE, Le KY, Tan VY, Duong AC, Chatterjee SS, Cheung GY, Joo HS, Hickok NJ, Otto M. 2015. Role of Phenol-Soluble Modulins in Formation of Staphylococcus aureus Biofilms in Synovial Fluid. Infect Immun 83:2966-75.

16. de Lorenzo V, Timmis KN. 1994. Analysis and construction of stable phenotypes in gram-negative bacteria with Tn5- and Tn10-derived minitransposons. Methods Enzymol 235:386-405.

17. Guyer MS, Reed RR, Steitz JA, Low KB. 1981. Identification of a sex-factor-affinity site in *E. coli* as gamma delta. Cold Spring Harb Symp Quant Biol 45 Pt 1:135-40.

18. Piewngam P, Zheng Y, Nguyen TH, Dickey SW, Joo HS, Villaruz AE, Glose KA, Fisher EL, Hunt RL, Li B, Chiou J, Pharkjaksu S, Khongthong S, Cheung GYC, Kiratisin P, Otto M. 2018. Pathogen elimination by probiotic *Bacillus* via signalling interference. Nature 562:532-537.

19. Fazli M, Harrison JJ, Gambino M, Givskov M, Tolker-Nielsen T. 2015. In-frame and unmarked gene deletions in *Burkholderia cenocepacia* via an allelic exchange system compatible with Gateway technology. Appl Environ Microbiol 81:3623-30.

20. Mlynek KD, Bulock LL, Stone CJ, Curran LJ, Sadykov MR, Bayles KW, Brinsmade SR. 2020. Genetic and biochemical analysis of CodY-mediated cell aggregation in *Staphylococcus aureus* reveals an interaction between extracellular DNA and polysaccharide in the extracellular matrix. J Bacteriol 202:e00593-19.

21. Choi KH, Schweizer HP. 2006. mini-Tn7 insertion in bacteria with single *att*Tn7 sites: example *Pseudomonas aeruginosa*. Nat Protoc 1:153-61.
