# Supplementary material for: Interspecies secreted surfactants induce emergent motility in Pseudomonas aeruginosa
Source: bioRxiv. 2024 Jan 4:2024.01.03.573969. Preprint. [Version 1] doi: 10.1101/2024.01.03.573969 (PMC10802355; doi:10.1101/2024.01.03.573969)
Supplement: Supplementary file 2 [file media-2.pdf]

EXTENDED FIGURES

TITLE

Interspecies secreted surfactants induce emergent motility in *Pseudomonas aeruginosa*

AUTHORS

Delayna L. Warrell[a]*, Tiffany M. Zarrella[a,b,c#]* , Christopher Machalek[a], Anupama Khare[a#]

[a]Laboratory of Molecular Biology, Center for Cancer Research, National Cancer Institute, National Institutes of Health, Bethesda, MD, USA

[b]Postdoctoral Research Associate Training Program, National Institute of General Medical Sciences, National Institutes of Health, Bethesda, MD, USA

[c]Current address: Department of Biology, Georgetown University, Washington, DC, USA

Running head: Surfactants induce motility in *P. aeruginosa*

[#]Address correspondence to Tiffany M. Zarrella, tiffany.zarrella@georgetown.edu, or Anupama Khare, anupama.khare@nih.gov

*Delayna L. Warrell and Tiffany M. Zarrella contributed equally to this work. Author order was determined alphabetically.

Key words: *Pseudomonas aeruginosa*, *Staphylococcus aureus*, surfactants, motility, polymicrobial interactions, phenol-soluble modulins

The authors declare no conflict of interest.

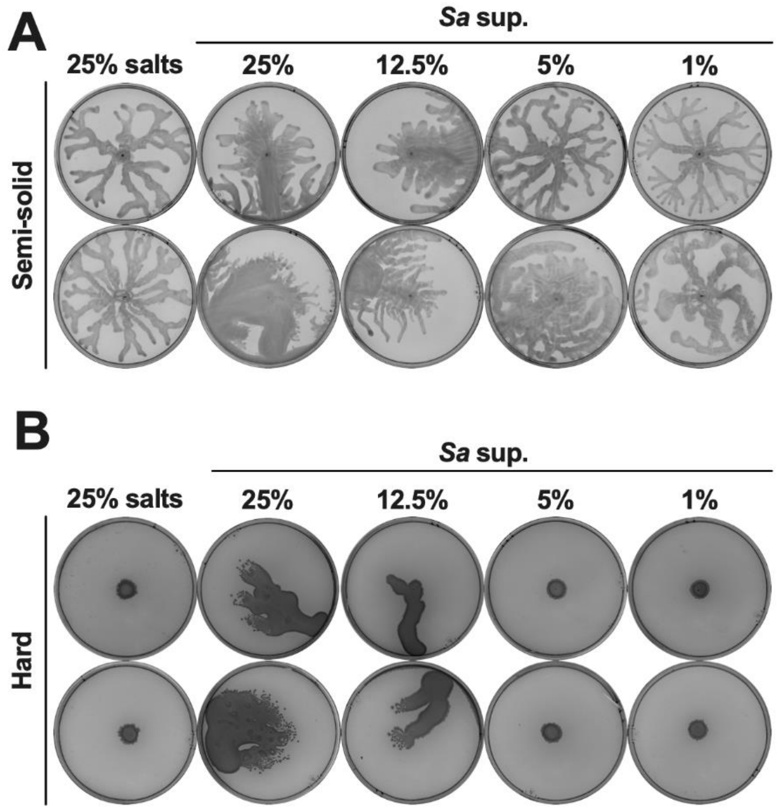

Extended Figure 1. *S. aureus* secreted products induce *P. aeruginosa* emergent motility in a dose-dependent manner. Additional replicates shown for Figure 1.

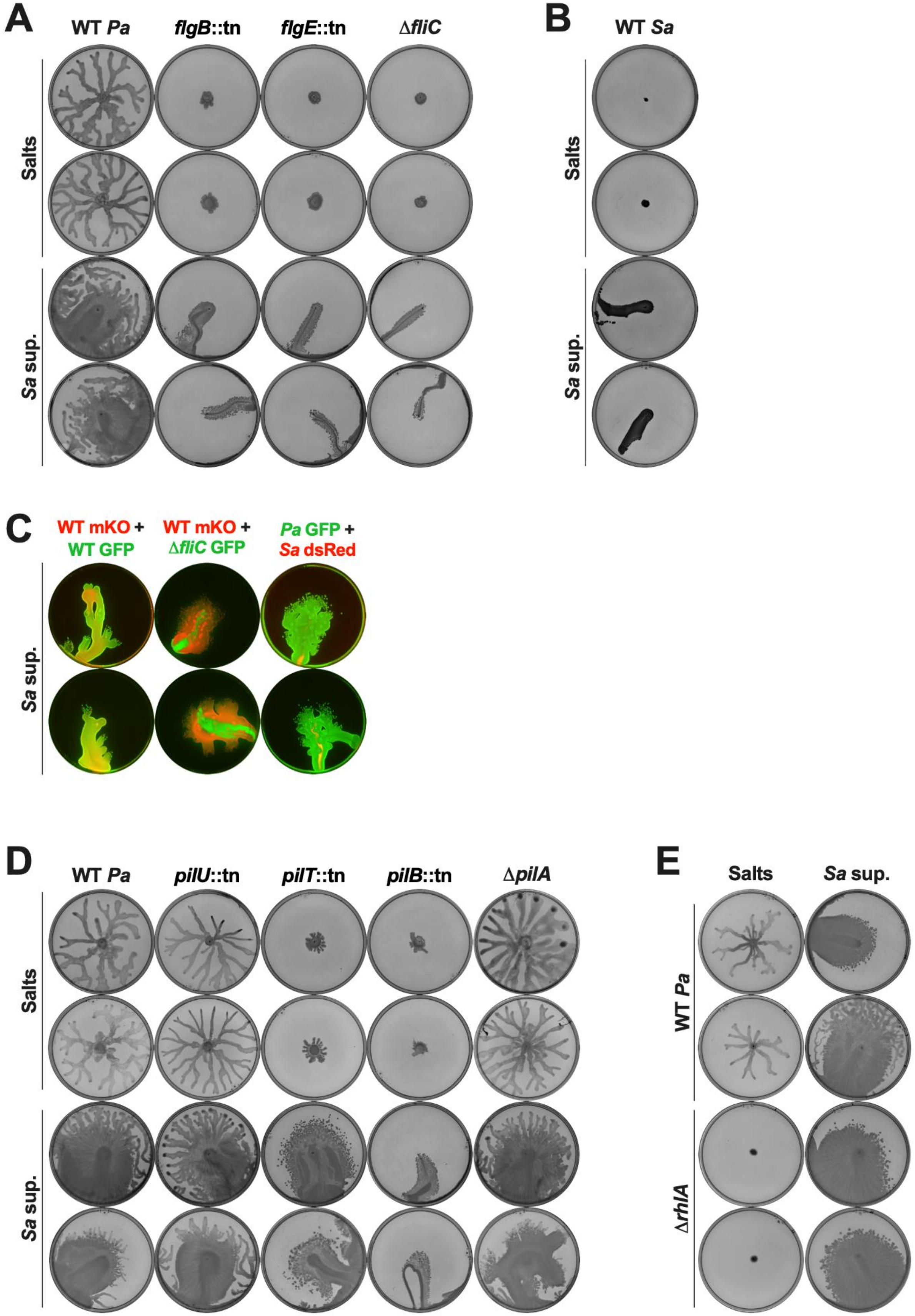

A
WT Pa
flgB::tn
flgE::tn
ΔfliC
Salts
Sa sup.
B
WT Sa
Salts
Sa sup.
C
WT mKO +
WT GFP
WT mKO +
ΔfliC GFP
Pa GFP +
Sa dsRed
Sa sup.
D
WT Pa
pilU::tn
pilT::tn
pilB::tn
ΔpilA
Salts
Sa sup.
E
Salts
Sa sup.
WT Pa
ΔrhlA

Extended Figure 2. Emergent motility requires flagella, but not pili or rhamnolipids.

Additional replicates shown for Figure 2.

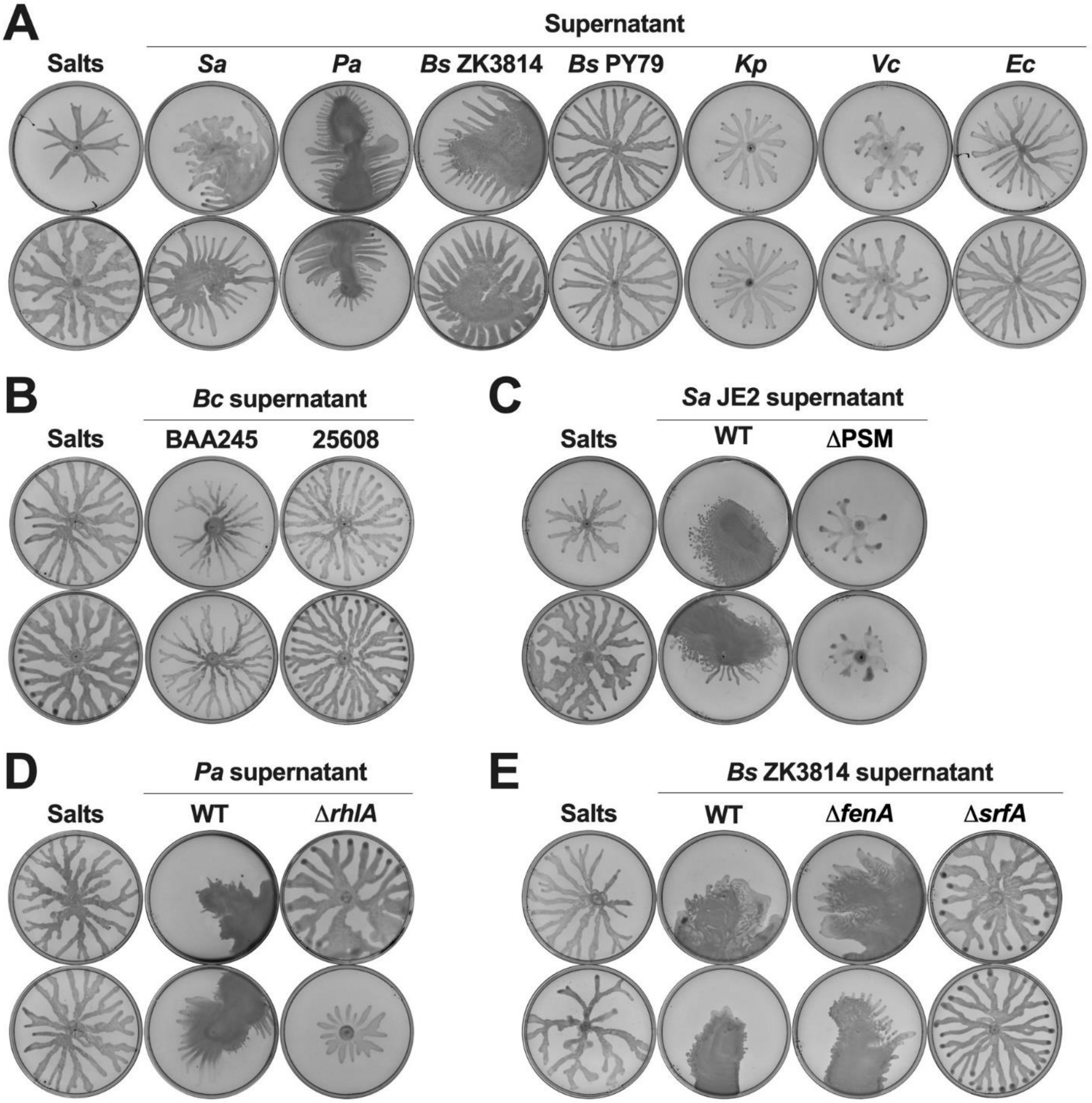

Extended Figure 4. Interspecies secreted surfactants induce emergent motility in *P. aeruginosa*. Additional replicates shown for Figure 4.

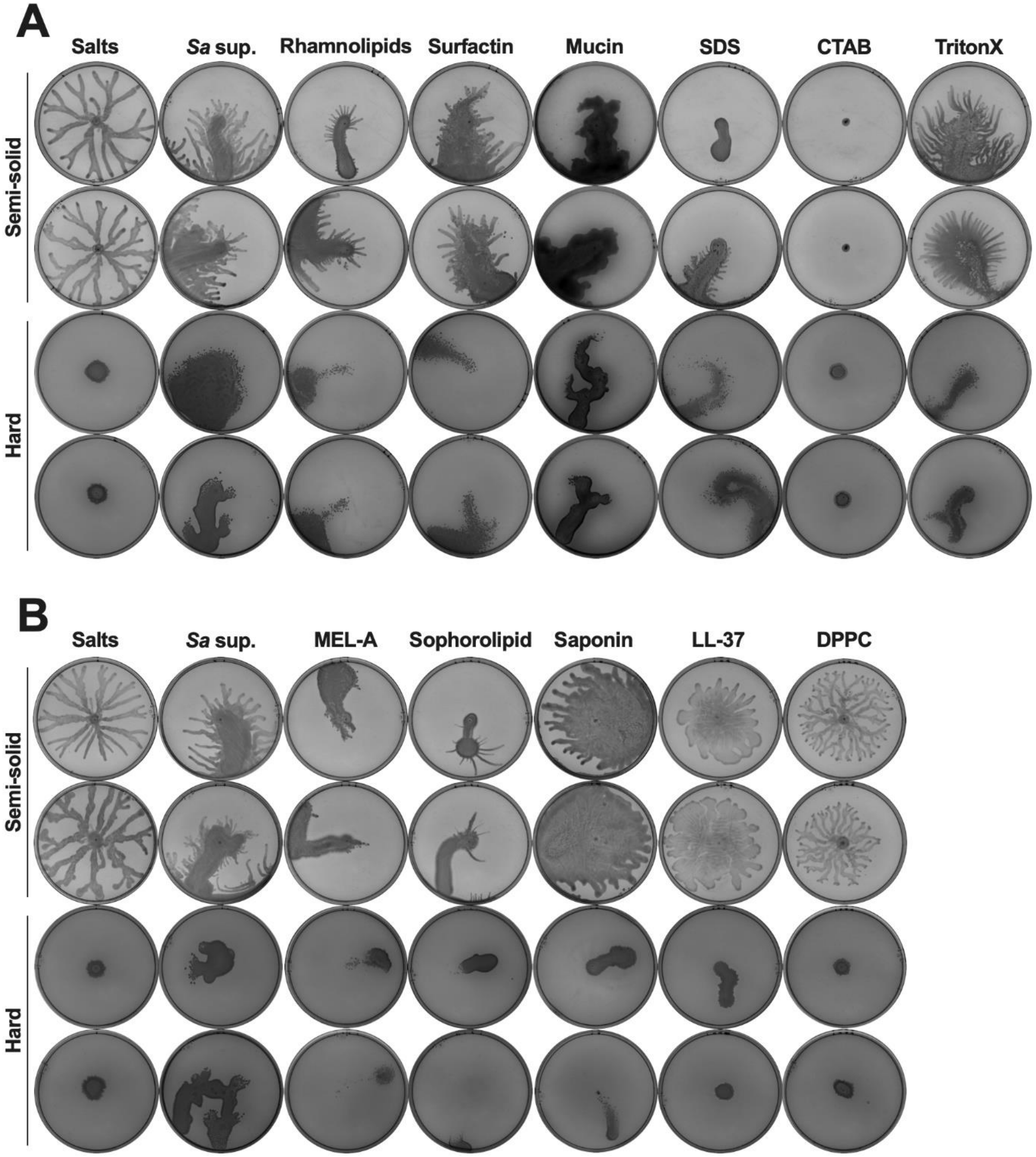

Extended Figure 5. The addition of diverse biotic and synthetic surfactants is sufficient to induce emergent motility in *P. aeruginosa*. Additional replicates shown for Figure 5.

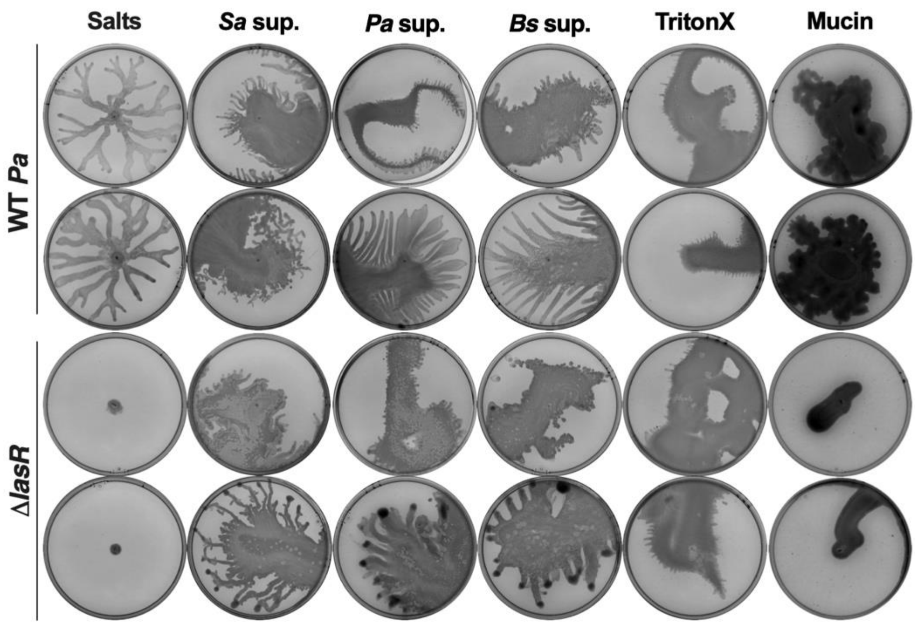

Extended Figure 6. The Las system is required for motility on mucin, but not on surfactants. Additional replicates shown for Figure 6.
